# Topological screen identifies hundreds of Cp190- and CTCF-dependent *Drosophila* chromatin insulator elements

**DOI:** 10.1126/sciadv.ade0090

**Published:** 2023-02-03

**Authors:** Tatyana G. Kahn, Mikhail Savitsky, Chikuan Kuong, Caroline Jacquier, Giacomo Cavalli, Jia-Ming Chang, Yuri B. Schwartz

**Affiliations:** ^1^ Department of Molecular Biology, Umeå University, Umeå, Sweden.; ^2^ Department of Computer Science, National Chengchi University, Taipei City, Taiwan.; ^3^ Institute of Human Genetics, UMR9002 CNRS, Montpellier, France.

## Abstract

*Drosophila* insulators were the first DNA elements found to regulate gene expression by delimiting chromatin contacts. We still do not know how many of them exist and what impact they have on the *Drosophila* genome folding. Contrary to vertebrates, there is no evidence that fly insulators block cohesin-mediated chromatin loop extrusion. Therefore, their mechanism of action remains uncertain. To bridge these gaps, we mapped chromatin contacts in *Drosophila* cells lacking the key insulator proteins CTCF and Cp190. With this approach, we found hundreds of insulator elements. Their study indicates that *Drosophila* insulators play a minor role in the overall genome folding but affect chromatin contacts locally at many loci. Our observations argue that Cp190 promotes cobinding of other insulator proteins and that the model, where *Drosophila* insulators block chromatin contacts by forming loops, needs revision. Our insulator catalog provides an important resource to study mechanisms of genome folding.

## Teaser

Topological screen identifies hundreds of Drosophila chromatin insulator elements and questions looping model of insulator action.

## INTRODUCTION

Eukaryotic chromosomes are extensively folded to fit inside micrometer-size cell nuclei. The degree of folding varies between different regions of the chromosome, and the specific folding patterns vary from cell to cell. Nevertheless, high-resolution imaging (*1*–*6*) and high-throughput chromosome conformation capture (Hi-C) assays (*7*–*9*) indicate that certain spatial conformations appear more frequently or persist longer than others. Averaged over large populations of cells, these conformations appear as submegabase-long chromatin regions, often referred to as topologically associating domains (TADs). Any two loci situated within such domain are more frequently in proximity than any two loci positioned in the two neighboring TADs (*10*, *11*).

What mechanisms cause the folding biases? To what extent do these biases influence gene expression? Both questions remain a subject of debate. Electrostatic interaction between nucleosomes, DNA supercoiling, chromatin loop extrusion by the cohesin complexes, and chromatin insulator elements were all proposed to play a role in shaping genome folding (*12*, *13*). Here, we will focus on chromatin insulators as they seem to have evolved for the regulation of gene expression.

These elements were first found in *Drosophila melanogaster* (*14*–*16*) but later identified in several developmental genes of flies and vertebrates (*17*–*27*). On the basis of transgenic experiments in *Drosophila*, it was proposed that chromatin insulators bias chromatin folding by interacting with each other (*28*, *29*). In this view, chromatin loops, formed by two or more interacting insulator elements, compete with contacts between chromatin sites inside and outside the loop. It was hypothesized that insulator-binding proteins equipped with protein-protein interaction domains hold the insulator elements together. The “bridging” proteins are recruited to insulator elements by auxiliary sequence-specific DNA binding proteins.

Consistently, a number of sequence-specific DNA binding proteins [CCCTC-binding factor (CTCF), Su(Hw), Ibf1, Ibf2, Pita, ZIPIC, BEAF-32, and M1BP] and two candidate bridging proteins [Cp190 or Mod(mdg4)] were implicated in *Drosophila* insulator function by genetic and biochemical screens [reviewed in (*30*)]. Of those, only one, CTCF, has a clear ortholog in vertebrates. As would be expected from the model, mammalian CTCF is frequently found at bases of chromatin loops detected by Hi-C. However, in this case, the correspondence is attributed to CTCF acting as a barrier for cohesin complexes extruding chromatin loops (*31*, *32*). This process does not require interactions between CTCF molecules bound at different insulator elements. Contrary to vertebrates, there is no evidence that *Drosophila* insulators block cohesin-mediated chromatin loop extrusion. Furthermore, Hi-C experiments in fly embryos and cultured cells identified very few chromatin loops that may be linked to insulator protein binding sites (*33*–*36*). Thus, to what extent the “looping model” applies to *Drosophila* insulators remains an open question.

How many fly genes are equipped with insulator elements is another question that proved difficult to address. Although *Drosophila* was the first multicellular organism where genomic distributions of multiple insulator proteins became available (*37*, *38*), this did not solve the problem. It turned out that the binding of individual proteins and even their combinations is a poor predictor of whether a site contains a functional insulator element (*38*, *39*). Therefore, only a couple of dozen *Drosophila* insulator elements have been characterized to date by transgenic assays that tested the blocking of enhancer-promoter communications or the spreading of a histone modification from a site tethering a histone methyltransferase [reviewed in (*30*, *40*)].

To close this gap, we undertook the parallel mapping of genomic contacts, transcriptomes, and genomic binding landscapes of insulator-binding proteins in cultured cells custom-derived from *Drosophila* embryos homozygous for the loss-of-function mutations in *CTCF* or *Cp190* genes. With this approach, we found hundreds of chromatin insulator elements. Their study indicates that chromatin insulators affect chromatin contacts locally at many individual loci and argues against the model where *Drosophila* insulators block chromatin contacts by forming insulator-insulator contacts.

## RESULTS

**Fig. 1.**
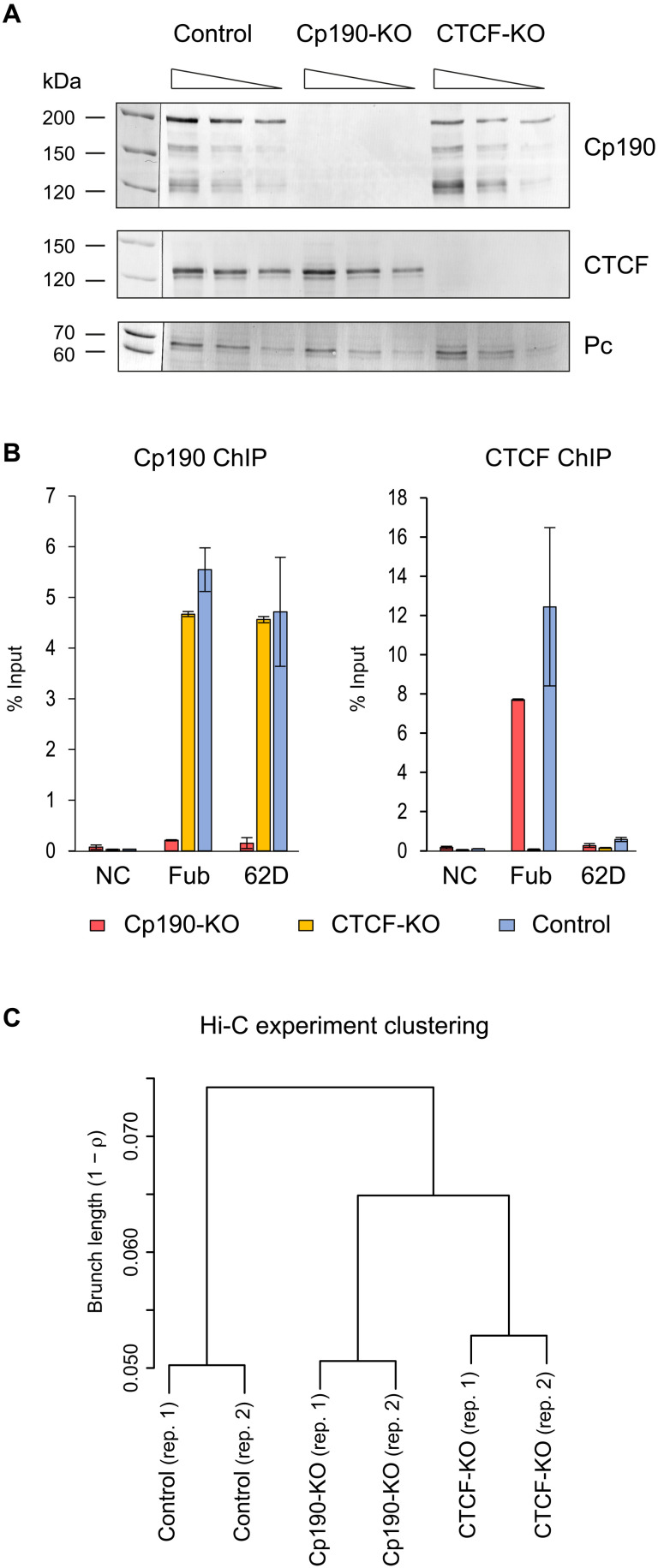
Characterization of Cp190- and CTCF-deficient cultured cell lines. (**A**) Twofold dilutions of total nuclear protein from control (Ras 3), Cp190-KO (CP-R6), and CTCF-KO (CTCF 19.7-1c) cells were analyzed by Western blot with antibodies against Cp190, CTCF, and Polycomb (Pc, loading control). Additional loading control, Coomassie-stained gel of the corresponding total nuclear protein samples, is shown on fig. S2. Positions of molecular weight markers (in kilodaltons) are indicated on the left. (**B**) ChIP-qPCR demonstrates that Cp190, normally present at *Fub* and *62D* insulators, is no longer detectable at these elements in Cp190-KO cells. Similarly, immunoprecipitation of *Fub* by anti-CTCF antibodies is abolished by CTCF-KO. Histograms show the average of two independent ChIP-qPCR experiments with whiskers indicating the scatter between individual measurements. An intergenic region from chromosome 3L that does not bind any insulator proteins was used as a negative control (NC). (**C**) Hierarchical clustering of Hi-C experiments based on pairwise Spearman correlation coefficients (average ρ for the group). To suppress spurious experimental noise, contacts within individual 40-kb bins (the diagonal of contact matrix) and contacts between bins separated by more than 1.6 Mb were excluded from calculations. For bins equal or larger than 40 kb, the clustering is robust to parameter changes (fig. S4).

To derive CTCF- and Cp190-deficient cells, we used the Ras^V12^ transformation approach (*41*) and embryos homozygous for *CTCF^y+1^* and *Cp190^3^* mutations (fig. S1). The *CTCF^y+1^* allele is a 3.3-kb deletion that removes the entire open reading frame of the *CTCF* gene and produces no protein (*39*, *42*). The *Cp190^3^* is a point mutation that results in premature translation termination at position Q61 and a short nonfunctional product (*43*). Although CTCF and Cp190 proteins are essential for fly viability, the mutant cells are viable and proliferate in culture. We derived two *Cp190^3^* and two *CTCF^y+1^* mutant cell lines. In all cases, the presence of the corresponding mutation was confirmed by polymerase chain reaction (PCR) genotyping (fig. S1B) and sequencing (fig. S1D) so we focused our analyses on Cp190-deficient line CP-R6 and CTCF-deficient line CTCF 19.7-1c, hereafter referred to as Cp190 knockout (Cp190-KO) and CTCF knockout (CTCF-KO) cells. Western blot analyses detected no Cp190 in the Cp190-KO or CTCF in the CTCF-KO cell lines (Fig. 1A), and chromatin immunoprecipitation coupled to quantitative PCR (ChIP-qPCR) analysis of previously characterized binding sites (*36*, *38*, *44*) confirmed the loss of Cp190 and CTCF from the chromatin of the corresponding mutant cells (Fig. 1B). Western blot assay indicates that the loss of CTCF does not cause a reduction in the overall Cp190 level, and inversely, the CTCF level is not affected by the *Cp190^3^* mutation (Fig. 1A). Similarly, the ablation of Cp190 or CTCF does not alter bulk levels of several other key insulator proteins tested (fig. S2). From this, we concluded that our mutant cell lines are a valuable system that provides large quantities of material to interrogate specific roles of Cp190 and CTCF in the three-dimensional genome organization.

### Cp190 loss and topological organization of the homeotic gene cluster

How does the loss of Cp190 or CTCF affect the three-dimensional conformation of the genome? To address this question, we used the Hi-C assay (*9*) to map chromatin contacts in CTCF-KO, Cp190-KO, and Ras^V12^ transformed but otherwise wild-type cells (Ras3, control) (*45*). After removing sequencing reads corresponding to circularized, unligated, or nondigested fragments, we detected from 44,406,664 to 81,238,455 chromatin contacts per each replicate and each genetic condition (for detailed statistics, see tables S1 and S2). Assigned to genomic segments of fixed size (Hi-C bins), contact frequencies measured in replicate experiments are highly correlated. Correlation progressively increases when bins of larger size are analyzed (from ρ = 0.81 to 0.88 for a small 5-kb bin to ρ = 0.98 to 0.99 for a 160-kb bin) and overall indicates that our Hi-C assay is reproducible. The contact frequencies remain strongly correlated when compared between different cell lines (fig. S3A), and, visualized at chromosome arm scale, contact maps of all three cell lines appear similar (fig. S3B). This argues that Cp190 or CTCF ablation does not grossly disrupt genome folding. Nevertheless, hierarchical clustering indicates that contact patterns in Cp190-KO and CTCF-KO cells are measurably distinct from those of the control cells (Fig. 1C and fig. S4).

**Fig. 2.**
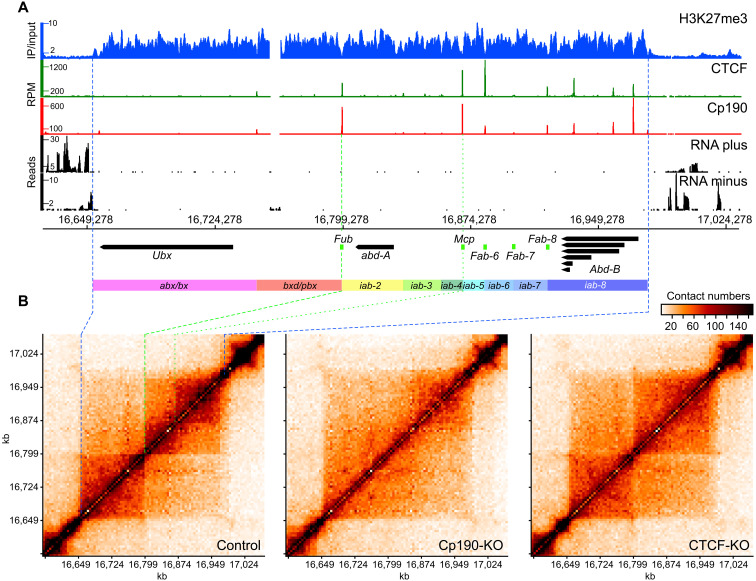
Organization and chromatin topology of the bithorax complex. (**A**) Genomic organization of the bithorax complex. ChIP-on-chip profiles of H3K27me3 in ML-DmBG3-c2 cells from (*52*) displayed as immunoprecipitation/input ratio, ChIP-seq profiles for Cp190 and CTCF in control cells [this study; displayed as the number of sequencing reads per position per million (RPM) of total reads], and RNA-seq profiles from control cells (displayed separately for each DNA strand as the number of sequencing reads per position) are shown above the coordinate scale (*dm6*, 2014 genome release). The positions of main alternative transcripts for *Ubx*, *abd-A*, and *Abd-B* genes are shown as thick arrows pointing in the direction of transcription. Note that transcripts flanking the bithorax complex genes are omitted for clarity. The positions of genetically defined insulator elements are indicated with green boxes. Regulatory domains are indicated as colored rectangles. (**B**) Chromatin contacts within the bithorax complex of the control, Cp190-KO, and CTCF-KO cells. The contacts measured by individual Hi-C experiments were assigned to 5-kb bins and normalized by IC (*90*). The data from replicate experiments were combined and plotted with gcMapExplorer software (*80*). The correspondence between the edges of the H3K27me3 domain in (A) and (B) is shown with blue dashed lines. The green dashed line indicates the position of the *Fub* insulator element, and the green dotted line shows the location of the *Mcp* insulator element.

To understand these differences, we started with a close inspection of the bithorax cluster of homeotic genes. The bithorax complex consists of three genes *Ubx*, *abd-A*, and *Abd-B* (Fig. 2A), which encode transcription factors responsible for segmental identity of the abdomen and posterior thorax (*46*). Correct segment-specific expression of *Ubx*, *abd-A*, and *Abd-B* is achieved by coordinated action of distal enhancers and Polycomb response elements (PREs). These enhancers and PREs are clustered in genetically defined domains (Fig. 2A). The domains *abx/bx* and *bxd/pbx* control the expression of *Ubx*. Series of *infra-abdominal* (*iab*) domains control the expression of *abd-A* (*iab-2*, *iab-3*, and *iab-4*) and *Abd-B* (*iab-5*, *iab-6*, *iab-7*, and *iab-8*) (*47*–*49*). Five known insulator elements (*Fub*, *Mcp*, *Fab-6*, *Fab-7*, and *Fab-8*) are required to ensure that the enhancers and PREs activate or repress the correct genes in correct body segments (*17*, *19*, *25*, *50*). Of these, *Fub* is exceptionally robust in that it can block enhancer-promoter interactions regardless of its location in the genome (*39*). The *Fub* insulator is required to prevent erroneous activation of *abd-A* by the *Ubx* enhancers (*19*, *51*).

In the control cells, *Ubx*, *abd-A*, and *Abd-B* are repressed by Polycomb mechanisms (*45*). Sequencing of total RNA [RNA sequencing (RNA-seq)] confirms that in these cells, all three genes are transcriptionally inactive (Fig. 2A). As illustrated in Fig. 2B, in the control cells, the bithorax complex is contained within a TAD whose borders match the borders of the chromatin domain enriched in histone H3 trimethylated at Lysine 27 (H3K27me3) (*52*, *53*). This domain is further split into two obvious large subdomains at a position precisely matching that of the *Fub* insulator element (Fig. 2, A and B). The outstanding enhancer-blocking activity of *Fub* requires Cp190 but not CTCF (*39*). This is because, in addition to CTCF, *Fub* contains recognition sequences for other DNA binding proteins including Su(Hw). The latter directly interacts with Cp190 and can tether Cp190 to *Fub* even when CTCF is absent (*51*). In perfect agreement with genetic and molecular data, in Cp190-KO cells, but not in the CTCF-KO cells, the topological boundary between the *Ubx* and *abd-A* genes disappears (Fig. 2B).

In addition, two distinct density clouds of chromatin contacts within *abd-A* and *Abd-B* genes and their regulatory regions are clearly visible in the contact map of the control cells (Fig. 2B, top right corner of the bithorax complex TAD). The clouds segregate at approximately the site of the *Mcp* insulator element (even though our Hi-C assay does not single out *Mcp* as a clear-cut boundary), and they are no longer visible in the contact maps from the Cp190-KO and CTCF-KO cells.

Three conclusions follow from the observations above. First, our experimental system is sufficiently sensitive and accurate to detect topological changes around robust enhancer-blocking insulators. However, it may miss those associated with weaker elements. Second, the *Fub* insulator is capable to limit chromatin contacts when the entire bithorax complex is transcriptionally inactive and repressed by Polycomb mechanisms. Last, our observations suggest that a systematic screen for Cp190- and CTCF-binding sites that limit chromatin contacts in the control cells but lose this ability in Cp190-KO and/or CTCF-KO cells may serve as a genome-wide approach to discover robust insulator elements.

### Genome-wide survey of *Drosophila* insulator elements

Insulator proteins bind the genome in distinct combinations (*38*, *54*), and some of these cobinding combinations correlate with an enhancer-blocking ability. Nevertheless, it is not possible to predict *Drosophila* insulator elements from genome-wide mapping data alone (*38*). As a first step to bridge this gap, we tested whether our strategy detects known insulator elements. About 30 *Drosophila* insulator elements, including those from the bithorax complex, have been identified by genetic assays to date. Only one of them, the *gypsy*-like *62D* insulator element from the intergenic region between the *ACXD* and *CG32301* genes, has a robust position-independent enhancer-blocking capacity comparable to that of the *Fub* (*44*, *55*). *62D* insulator binds Su(Hw), Cp190, and Mod(mdg4) but not CTCF (Fig. 1B) (*44*, *55*). Consistently, in the control and CTCF-KO cells, the position of the *62D* insulator element coincides with a point of reduced contact crossing, which is alleviated in Cp190-KO cells (fig. S5).

**Fig. 3.**
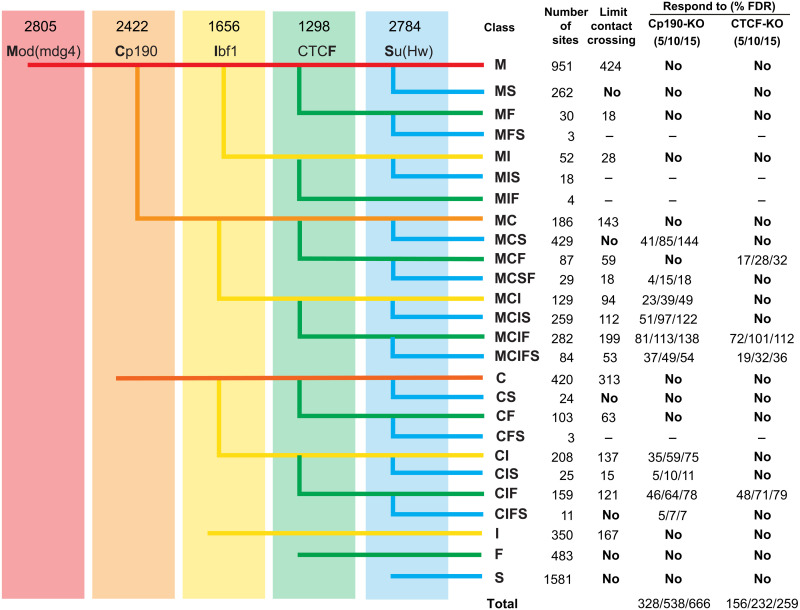
Classes of insulator protein binding regions. Genomic positions of regions enriched by immunoprecipitation of the chromatin from control cells (numbers indicated above each antibody) were compared pairwise in order from left to right. Thus, regions enriched by ChIP with antibodies against Mod(mdg4) were checked for overlap with regions enriched with antibodies against Cp190. The resulting three groups, i.e., bound by both Mod(mdg4) and Cp190, bound by just Mod(mdg4), or bound by just Cp190, were further compared to regions enriched by ChIP with antibodies against Ibf and so on. The resulting cobinding classes were designated with combinations of single letters representing the individual insulator proteins present. Columns to the right indicate the number of regions within each class. For classes whose fraction of regions above the corresponding threshold significantly exceeds that of the control, the number of regions that limit contact crossing (γ > 75% of that for the control regions) or the number of regions that display an increase in the contact crossing [Δγ ≤ FDR (false discovery rate)] upon Cp190-KO or CTCF-KO is shown further to the right. MFS, MIS, MIF, and CFS sites were too few and therefore excluded from analyses.

Encouraged by this observation, we mapped the genomic binding of Cp190, CTCF, and several other key insulator proteins: Su(Hw), Mod(mdg4), and Ibf1 in the control and CTCF-KO and Cp190-KO cells using ChIP coupled to sequencing of the precipitated DNA (ChIP-seq). We performed two ChIP-seq experiments for each genetic background using independently prepared chromatins and had the DNA from the corresponding chromatin input materials sequenced to control for possible sample processing biases. We then used Model-based Analysis of ChIP-seq (MACS) algorithm (*56*) to identify genomic regions significantly enriched by immunoprecipitation with individual antibodies in the control cells and compared their genomic positions pairwise as illustrated in Fig. 3. This approach grouped all enriched regions according to 26 common cobinding patterns (cobinding classes), which we designated with combinations of single letters that represent individual insulator proteins. For example, regions cobound by Mod(mdg4), Cp190, Ibf1, CTCF, and Su(Hw) were designated as MCIFS.

How many regions in each cobinding class restrict chromosomal contacts? What fraction of those cease to limit contacts in Cp190-KO and/or CTCF-KO cells? To address these questions, we used Hi-C measurements to calculate the propensity of the chromatin contacts to cross insulator protein bound regionsin the three cell lines. The frequency with which interaction between any two chromosomal sites is captured by Hi-C decays exponentially with increasing genomic distance between the sites (*9*, *57*, *58*). The global decay in contact frequency as a function of genomic distance is similar for all chromosome arms and can be approximated by a power law with a scaling exponent derived from Hi-C measurements (*9*, *32*). For most chromosomal sites, the frequency of pairwise interactions follows the global decay model and is, therefore, predictable from the genomic distance between them. However, if two sites are separated by a region that limits chromosomal contacts, the observed interaction frequency is lower than that predicted by the global decay model. The prediction is improved by fitting a distance-scaling factor (γ) to each restriction fragment assayed in Hi-C (*9*). In this approach, regions that limit chromosomal contacts are assigned high distance-scaling factors. Using a computational pipeline developed by Yaffe and colleagues (*9*), we calculated γ for restriction fragments participating in the Hi-C assay and assigned each insulator protein bound region the highest γ from all restriction fragments overlapped by that region.

**Fig. 4.**
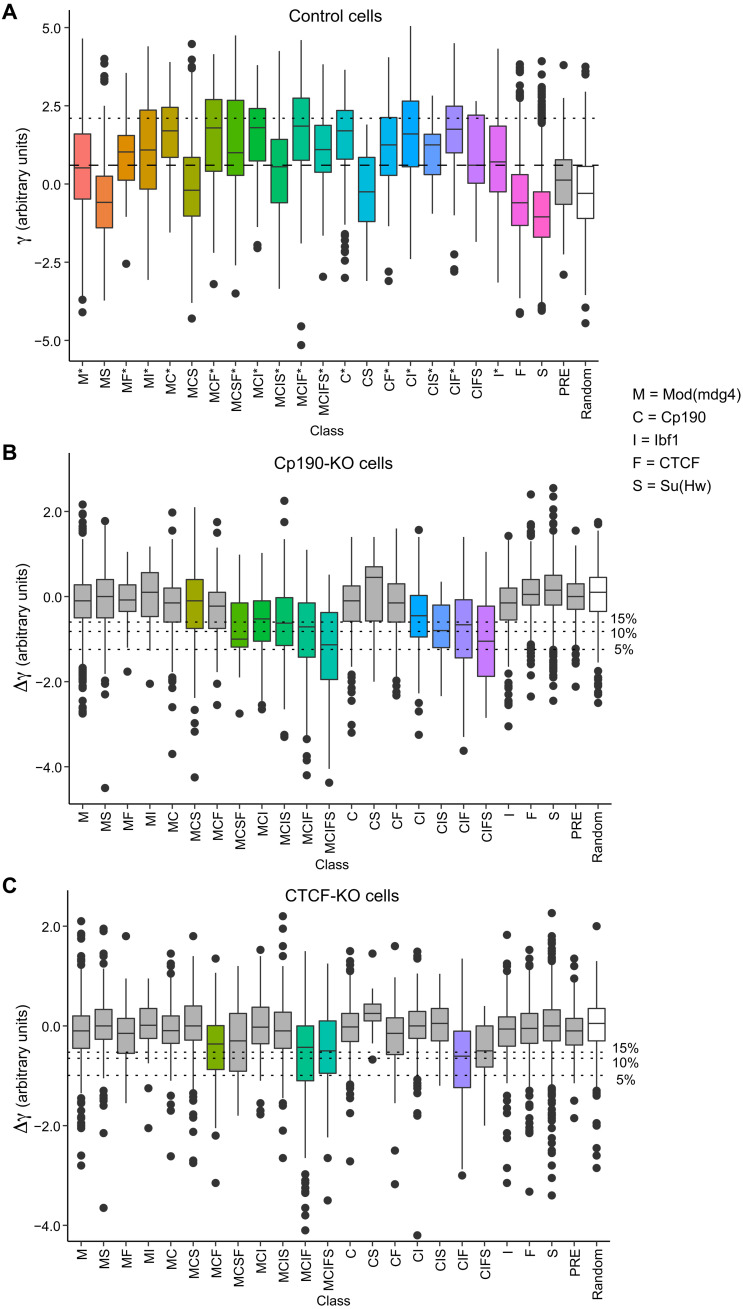
Specific combinations of insulator proteins limit chromatin contacts. (**A**) Box plots display distance-scaling factors (γ) at different classes of insulator protein binding sites (colored boxes), PREs (gray box), and control regions (random, white box). Here and in all subsequent figures, the box plots indicate the median and span interquartile range with whiskers extending 1.5 times the range and outliers shown as black dots. Sites with γ above the top quartile in the control group (horizontal dashed line) are considered as limiting chromatin contact crossing. Classes for which the fraction of such sites is significantly greater than that in the control group (*P* < 0.0001, one-tailed Fisher’s exact test) are marked with asterisks. Horizontal dotted line indicates the top 5% value for γ genome-wide used to define TAD borders by Sexton and colleagues (*9*). KO of Cp190 (**B**) and CTCF (**C**) leads to systematic reduction of distance-scaling factors (negative Δγ = γ_KO_ − γ_control_) at some classes of insulator protein binding sites. The control group was used to define FDRs (% FDR, horizontal dotted lines). Classes for which the fraction of sites with Δγ below the 10% FDR cutoff significantly exceeds that in the control group (*P* < 0.0001, one-tailed Fisher’s exact test) are marked with color.

As illustrated in Fig. 4A, some classes of insulator protein bound regions tend to limit contact crossing (tend to have high γ), while others are no different from randomly chosen control regions that do not bind insulator proteins. As noted previously (*38*, *54*), the classes that tend to limit contact crossing tend to cobind multiple insulator proteins. However, the simple binding of large protein complexes does not explain the effect. For example, PREs, which bind megadalton-size Polycomb complexes, have a distribution of γ similar to that of the control regions (Fig. 4A). Consistent with previous transgenic enhancer-blocking tests (*38*), regions that bind CTCF but no Mod(mdg4) or Cp190 (“standalone” F class) do not limit contact crossing and have low γ. This indicates that *Drosophila* CTCF requires additional partners to affect the chromatin topology.

The 95th percentile for γ genome-wide has been used as a threshold to define TAD borders (*9*). By this criterion, there are 1008 TAD borders in the control cells consistently identified in both replicate experiments (fig. S6). Of those, 913 correspond to one of the insulator protein bound regions, which means that the majority (90.6%) of the TAD borders bind one or more known insulator proteins. In contrast, only 14.8% of all insulator protein bound regions are identified as TAD borders, although many of the insulator protein bound regions not identified as TAD borders are harder for chromatin contacts to cross compared to the control group (Fig. 4A). These observations illustrate that it is difficult to pick a γ-based threshold that will reliably single out chromatin insulator elements.

Instead, we sought to identify insulator protein binding regions over which the chromatin contacts increase in Cp190-KO or CTCF-KO cells. The change is described by the difference between distance-scaling factors calculated from the Hi-C measurements in mutant and control cells (Δγ = γ_KO_ − γ_control_). As expected, the median Δγ for the control (random) regions is close to zero for both Cp190-KO and CTCF-KO (Fig. 4, B and C), although, at some of the regions, Δγ deviates because of technical variability of Hi-C as well as inherent variability of cultured cell lines. In contrast, several classes of insulator protein bound regions show a systematic increase in chromatin contact crossing (negative Δγ) upon Cp190-KO or CTCF-KO (*P* < 0.0001, one-sided Fisher’s exact test; Fig. 4, B and C). The regions that, in wild-type cells, bind Cp190 but no CTCF (e.g., MCS, MCI, MCIS, and CIS) become systematically easier to cross (negative Δγ) only in the Cp190-KO (Fig. 4B) but not in the CTCF-KO cells (Fig. 4C). This indicates that our assay is specific.

To single out putative chromatin insulator elements, we followed a two-step algorithm. First, using the distribution of Δγ values for the random control regions, we defined 5, 10, and 15% false discovery rate (FDR) thresholds. Second, using these thresholds, we selected all regions with Δγ ≤ FDR from classes that have a significantly higher fraction of regions with increased chromatin contact crossing in the corresponding mutant cells (marked with color in Fig. 4, B and C; see Materials and Methods for calculations). This way, we detected 745 putative insulator elements that require Cp190 or CTCF or both at 15% FDR (632 at 10% FDR; 401 at 5% FDR). For additional statistics and the list of elements, see Fig. 3 and table S3. This catalog includes *Fub*, *62D*, *1A2*, *SF1*, and *Homie* insulator elements identified by genetic assays (*19*, *22*, *39*, *44*, *55*, *59*–*61*). Approximately one-third of the insulators from our catalog (from 28.99% of the elements defined at 15% FDR to 30.67% defined at 5% FDR) reside within 2 kb from their nearest TAD border.

### Other factors that impair chromatin contact crossing

**Fig. 5.**
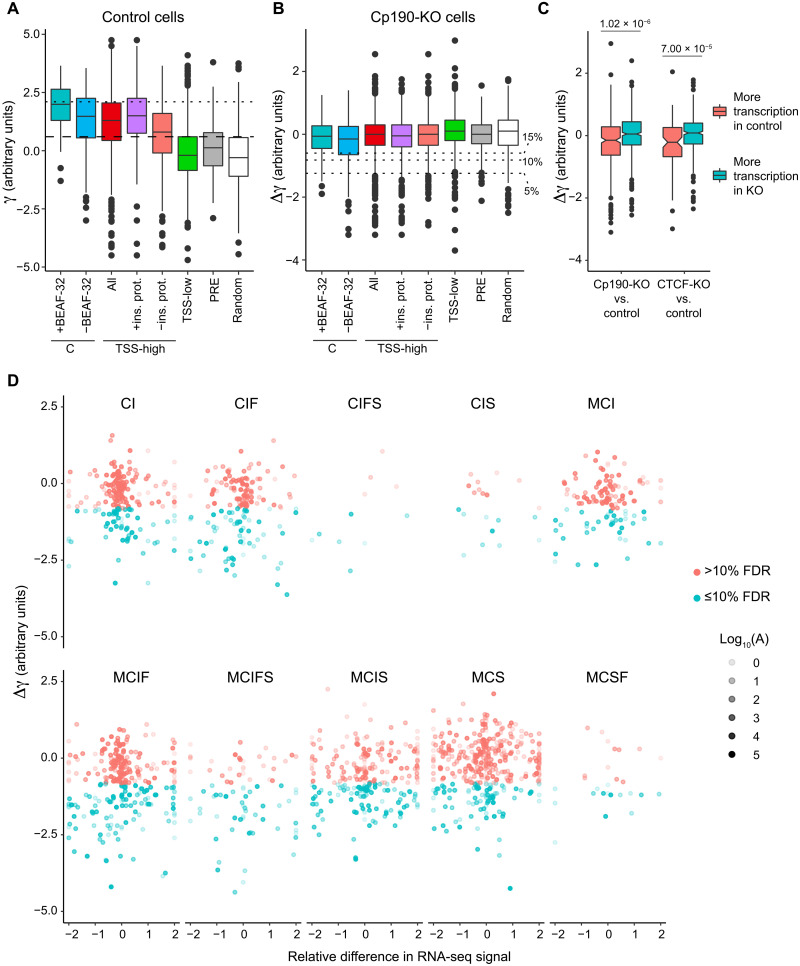
Transcription start sites of transcriptionally active genes impair chromatin contact crossing. (**A**) Box plots display distance-scaling factors (γ) in control cells at class C regions either cobound or not bound by BEAF-32. Also shown are γ values of transcription start site (TSS) grouped by transcriptional activity of the corresponding gene (TSS-high/all and TSS-low) and the binding of insulator proteins mapped in this study (TSS-high/+ins. prot and TSS-high/−ins. prot). Distance-scaling factors of PREs and randomly selected regions with no appreciable ChIP-seq signal for any of the insulator proteins are shown for comparison. Sites with γ above the top quartile in the random control group (horizontal dashed line) are considered as limiting chromatin contact crossing. Horizontal dotted line indicates the top 5% value for γ genome-wide. (**B**) Cp190 KO leads to no systematic change in distance-scaling factors (Δγ = γ_KO_ − γ_control_) at any of the classes of regions. Horizontal dotted lines indicate FDRs used to define insulator elements in Fig. 4. (**C**) Box plots of Δγ for TSSs of genes whose transcription differs (|log_2_ fold change| > 2) between Cp190-KO or CTCF-KO and control cells. Note the significant difference between Δγ for TSS with higher transcription in the mutant cells compared to that of the genes with higher transcription in the control cells (*P* values from Wilcoxon rank sum test are shown above). (**D**) Scatterplots compare the changes in distance-scaling factors (Δγ ≤ 10% FDR colored in cyan; Δγ > 10% FDR colored in pink) at insulator protein binding sites of various classes to relative differences in RNA-seq signals of genes closest to these sites after Cp190 KO. The average RNA-seq signals between control and Cp190-KO cells (A) in log_10_ scale are indicated by variable point intensities.

Most classes of insulator protein bound regions, which show an increase in chromatin contact crossing in the mutant cells (Δγ < 0), are hard to cross (have high γ) in the control cells (Fig. 4). However, the inverse is not true. For example, regions of the C, MC, and CF classes bind Cp190 and have high γ but show no increase in contact crossing upon Cp190 KO (Fig. 4). At these regions, other features can substitute for Cp190 or are the primary cause for the reduced chromatin contact crossing. What could these features be? BEAF-32 protein was implicated not only in the function of the *scs*’ insulator element (*62*) but also in transcriptional activation (*63*). Many of the BEAF-32–binding sites overlap Cp190-bound regions (fig. S7A) (*38*). Nevertheless, when we compare the class C regions that bind BEAF-32 to those that do not bind the protein, it is evident that BEAF-32 is not the primary cause of high γ at these regions (Fig. 5, A and B).

Further inspection of the C class indicates that most of these regions reside close to transcription start sites (TSSs) with a median distance of just 226 base pairs (bp) (fig. S7B). Sequencing of the total RNA indicates that most of the corresponding TSS belong to transcriptionally active genes (fig. S7C). Conceivably, proteins associated with transcription or spatial interactions between transcriptionally active genes restrict chromatin contact crossing regardless of Cp190 binding. The Hi-C bins encompassing “highly transcriptionally active” TSS (top quartile of the RNA-seq signals) have γ comparable to that of the C class (Fig. 5A). Many insulator protein bound regions are located close to transcriptionally active TSS (fig. S7, B and C), possibly contributing to the high γ of the latter. Nevertheless, TSSs of highly transcribed genes that have no Mod(mdg4), Cp190, Ibf1, CTCF, Su(Hw), or BEAF-32 bound within 1-kb distance are still hard for chromatin contacts to cross (Fig. 5A). Supporting this notion, chromatin contacts across TSSs whose transcription differs between Cp190-KO (or CTCF-KO) and control are harder to establish in cells where the corresponding genes are more transcriptionally active (Fig. 5C).

Two conclusions follow from the observations above. First, transcriptionally active genes hinder chromatin contacts regardless of their association with Cp190 or any other insulator protein tested here. This may be due to binding of other, possibly undiscovered, insulator proteins or spatial segregation of transcriptionally active genes. Second, a previously recognized link between Cp190 and TAD boundaries (*9*, *64*) should be interpreted with caution as a large fraction of Cp190-bound regions reside next to the TSS of transcriptionally active genes.

### Altered transcription and changes in chromatin contacts across insulator elements

Since transcriptionally active genes hinder the chromatin contact crossing, we wondered to what extent the increased contacts across insulator protein binding sites observed in Cp190- and CTCF-KO cells might have been caused by changes in transcription of the nearby genes. Using the DESeq2 algorithm (*65*), we identified 831 genes differentially transcribed in Cp190-KO cells compared to control cells, 475 genes differentially transcribed in CTCF-KO cells compared to control, and 691 genes differentially transcribed between Cp190-KO and CTCF-KO cells. Principal components analysis (PCA) indicates that the three cell lines have distinct changes in gene transcription, with Cp190-KO cells being slightly more different from either CTCF-KO or control cells (fig. S8A). We found no coherent transcriptional changes in Cp190-KO and CTCF-KO cells around insulator elements impaired in both cell lines, which would be expected if the changes have caused increased chromatin contacts across these sites. As illustrated by clustering of the top 20 most differentially transcribed genes (fig. S8B), some of the observed variability is due to the stochastic nature of cell line derivation and distinct genetic backgrounds of parental fly lines. For example, note that *yellow* (*y*), the gene most “up-regulated” in CTCF-KO cells (fig. S8B), comes from the transgene inserted in the *CTCF^y+1^* allele (*39*, *42*).

We see no correlation between the changes of distance-scaling factor (Δγ) at insulator protein binding sites and the transcription from the closest TSSs (Fig. 5D and fig. S9). Together, these observations argue that the changes in transcriptional activity of the nearby genes are not responsible for the increased contacts across the insulator protein binding sites observed in Cp190-KO and CTCF-KO cells. Instead, they are the direct consequence of the disrupted function of the underlying insulator elements.

### Chromatin insulator mode of action

**Fig. 6.**
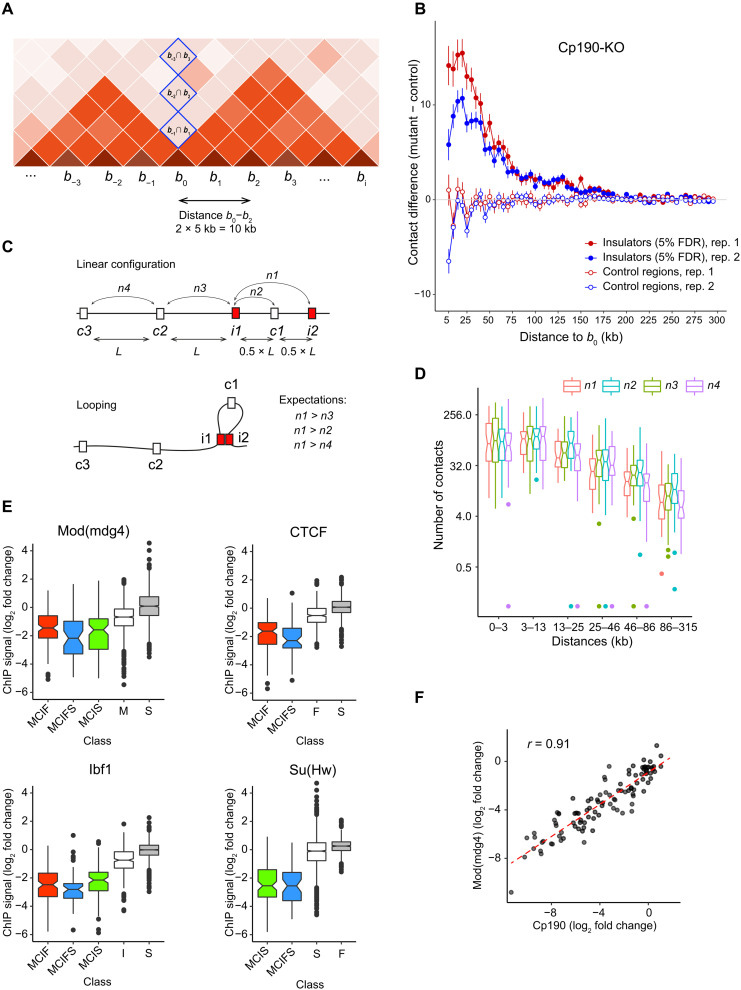
Action range and looping test. (**A**) Schematic representation of a chromatin contact matrix. Higher color intensity indicates more contacts. Blue rectangles mark matrix elements used to estimate contacts across a specific region (*b*_0_; see the main text for details). (**B**) Average contact crossing difference curves for Cp190-dependent insulator elements (filled circles) and control regions (empty circles) determined from two replicate experiments. Note that, at close distances (5 to 10 kb), estimates of chromatin contact frequencies become less reliable. (**C**) Schematic illustration of the insulator looping test. *L* designates the distance between a pair of the closest insulator elements (i1 and i2). (**D**) Pairs of the closest Hi-C bins containing insulator elements (defined at 15% FDR) were split in groups of equal sizes depending on their separation in the linear genome. The number of contacts between the paired insulators (*n1*) was plotted (red box plots) alongside the number of contacts between corresponding pairs of control regions [blue (*n2*), green (*n3*), and purple (*n4*) box plots]. Notches mark 95% confidence intervals of the medians. (**E**) Box plots of log_2_(Cp190-KO/control) changes in ChIP-seq signals at MCIF (red), MCIFS (blue), and MCIS (green) insulator sites. These are compared to standalone sites for the protein of interest (white) and the background noise at sites not significantly enriched by this protein (gray). No overlap between the box plot notches indicates that their medians are significantly different. (**F**) Scatterplot of log_2_(CTCF-KO/control) changes of ChIP-seq signals for Cp190 and Mod(mdg4) at MCIF insulators. Dashed red line shows the linear regression fit.

The extensive catalog of putative insulators may yield mechanistic insights into their function. As the first step toward this aim, we asked: From what distance could the contacts across insulator elements be blocked? To this effect, we calculated the number of contacts between pairs of Hi-C bins around each putative insulator starting from the two bins immediately adjacent to the insulator and followed by pairs at progressively larger distances (Fig. 6A). We then subtracted the values for the corresponding bin pairs calculated for mutant and control cells

$$\left(b_{-1}^{\mathrm{mut}}\cap b_{1}^{\mathrm{mut}}-b_{-1}^{\mathrm{con}}\cap b_{1}^{\mathrm{con}}),(b_{-2}^{\mathrm{mut}}\cap b_{2}^{\mathrm{mut}}-b_{-2}^{\mathrm{con}}\cap b_{2}^{\mathrm{con}})\ldots (b_{-i}^{\mathrm{mut}}\cap b_{i}^{\mathrm{mut}}-b_{-i}^{\mathrm{con}}\cap b_{i}^{\mathrm{con}}\right)$$

The resulting contact crossing difference values were averaged for all putative insulator elements to yield the cumulative insulator contact crossing difference curves (Fig. 6B and fig. S10). To control for potential sampling and normalization biases, we applied the same procedure to a set of randomly chosen regions that do not bind any of the insulator proteins. As expected, the cumulative contact crossing difference curves for control regions fluctuate around zero (Fig. 6B and fig. S10). In contrast, the curves for the putative insulator elements are positive up to the distances of ~150 kb (Fig. 6B and fig. S10). This argues that an average *Drosophila* insulator element can interfere with contacts between chromosomal sites that are up to 300 kb apart.

How *Drosophila* insulator elements interfere with chromatin contacts is not well understood. The most popular hypothesis suggests that fly insulators physically interact with each other and form chromatin loops, which, in turn, compete with chromatin contacts between chromosomal elements outside the loops. With an extensive catalog of insulator elements at hand, we sought to evaluate this hypothesis using a “looping test” illustrated in Fig. 6C. For each pair of the closest insulator elements from our catalog, we calculated the number of contacts between these insulators and between three matched control pairs. The first control pair consisted of one of the insulators (i1) and the control region (c1) halfway toward the second insulator (i2). The second control pair consisted of the insulators (i1) and the control region (c2) located at the same distance as the two insulators. Last, the third control pair included control regions c2 and c3 located at the same distance as the insulators. If insulator elements tend to interact with each other and form loops, we expect the number of contacts between the closest insulator pairs to be greater than that between the control pairs (*n1* > *n4*; *n1* > *n3*; *n1* > *n2*). The box plots of contact numbers (Fig. 6D) indicate that, regardless of genomic distances between the closest insulators, this is not the case.

The existing evidence for insulator interactions is largely derived from analyses of the elements incorporated into transgenic constructs. For example, certain insulators, when paired with themselves, become inefficient in blocking the transcriptional activation of a reporter gene by a remote enhancer (so called “insulator bypass”) (*28*, *29*, *66*). In these transgenic assays, insulators reside in close proximity, typically less than 5 kb apart. Our looping test estimates chromatin contact frequencies from proximity ligation (the underlying principle of the Hi-C method). These estimates become less reliable at short distances as the Hi-C resolution is limited by the size of DNA fragments generated after digesting the cross-linked chromatin with a restriction endonuclease. Conceivably, closely spaced insulators form loops that our Hi-C analysis did not detect. To address this issue, we repeated our looping test using the high-resolution Micro-C data from 2-hour-old wild-type (*y^1^w^67c23^*) embryos recently published by Batut and coauthors (*33*). The outcome of the test indicates that the insulators do not form more contacts than control pairs (fig. S11), just as in the case of our own Hi-C data. To summarize, the results of our tests provide no support for the model where *Drosophila* insulator elements block chromatin contacts by forming chromatin loops.

One may expect that sites bound by multiple insulator proteins would impair chromatin contacts even when Cp190 or CTCF is missing because the other proteins would compensate for their loss. Our experiments indicate the opposite (note the low Δγ for MCIF, MCIFS, and MCIS sites; Fig. 4, B and C). Two not mutually exclusive explanations may account for this. First, the ablation of Cp190 or CTCF may lead to the loss of other insulator proteins from these sites. Second, at sites cobound by multiple insulator proteins, the simultaneous presence of all proteins may be required to limit the chromatin contact crossing. To evaluate these possibilities, we compared the ChIP-seq signals for Mod(mdg4), Ibf, CTCF, and Su(Hw) at MCIF, MCIFS, and MCIS insulators between the Cp190-KO and control cells. For all proteins, the immunoprecipitation of these regions from the Cp190-KO chromatin is significantly reduced compared to that of the controls (Fig. 6E). This indicates that Mod(mdg4), Ibf, CTCF, or Su(Hw) requires Cp190 for efficient binding to MCIF, MCIFS, and MCIS sites. The CTCF-KO also affects Cp190 and Mod(mdg4) binding to the MCIF insulators to an extent that varies between individual sites. Further strengthening the Cp190 dependence argument, the reduction of Cp190 and Mod(Mdg4) ChIP-seq signals upon CTCF ablation is highly correlated (Fig. 6F). To summarize, it appears that the loss of Cp190, by mutation or due to impaired tethering by CTCF, impairs the binding of companion insulator proteins, which explains why these proteins do not compensate for Cp190 loss.

## DISCUSSION

Three main conclusions follow from our study. First, the *D. melanogaster* genome contains hundreds of Cp190- and/or CTCF-dependent chromatin insulators. While they appear to play a relatively minor role in shaping the overall chromosome folding patterns, they have a distinct impact on chromatin contacts at many specific loci. Second, we find that TSSs of transcriptionally active genes are generally hard for chromatin contacts to cross regardless of their association with Cp190 or any other insulator protein that we tested. Since many Cp190-bound regions reside next to the TSS of transcriptionally active genes, a previously recognized link between Cp190 and TAD boundaries should be interpreted with caution. Third, the expanded catalog of insulator elements is instrumental to advance our understanding of how these elements affect chromatin contacts. For example, we found no evidence that Cp190- or CTCF-dependent insulators preferentially interact with each other. This argues that new models, which do not invoke chromatin loops formed by interacting insulator elements, are required to explain the prevailing mechanism of insulator action. Broadly similar conclusions were reached by the study of Kaushal and coauthors (*67*) who reported the analysis of genome folding in *Drosophila* embryos deficient for Cp190 and CTCF while this work was being prepared for publication.

Found some 30 years ago, chromatin insulators were hailed as major players organizing the *Drosophila* genome into topologically independent regulatory units. This outlook started to fade as we learned more about the architecture of the fly chromatin. It became apparent that transcriptional activity is a better predictor of the overall *Drosophila* TAD organization than the genomic distribution of insulator proteins (*68*) and that partial depletion of insulator proteins by RNA interference causes only limited changes in patterns of posttranslational histone modifications or gene transcription (*38*, *69*). It was then hypothesized that topological partitioning of the *Drosophila* genome is driven primarily by interactions between transcriptionally active genes (*36*, *68*) and that, because of its much more compact genome, flies may not require additional mechanisms to regulate genome architecture (*36*, *70*). Consistent with this view and in contrast to vertebrates, there is no evidence that *Drosophila* TADs arise from blocking cohesin-mediated chromatin loop extrusion.

Our work reconciles the two assessments. On one hand, our Hi-C analyses indicate that complete ablation of Cp190 or CTCF does not grossly disrupt the genome folding. On the other hand, we show that *Drosophila* genome contains more than 700 putative insulator elements, which restrict chromatin contact crossing and can interfere with contacts between chromosomal sites up to 300 kb apart. As we know from prior genetic analyses, some of these insulators, e.g., *Fub*, are essential for the correct regulation of developmental genes. These insulator elements restrict chromatin contacts regardless of transcriptional activity within the neighboring chromatin. As exemplified by *Fub*, they can impair chromatin contacts within a locus repressed by the Polycomb mechanisms. While transcription-related mechanisms appear to define the major contact patterns within the *Drosophila* genome, insulators have widespread but shorter-range impact.

As clear from observations presented here and those by Kaushal and coauthors (*67*), at many sites cobound by CTCF and Cp190, the former contributes to Cp190 recruitment to chromatin. However, ChIP-seq analysis of sites bound by multiple insulator proteins (e.g., of MCIF, MCIFS, and MCIS classes) indicates that, at these locations, Cp190 is required for efficient binding of other insulator proteins. Unexpectedly, those include the sequence-specific DNA binding proteins CTCF and Su(Hw). Three not mutually exclusive possibilities can account for this observation. First, it is possible that the Zn-finger domains of Cp190 increase the overall affinity of the multiprotein insulator complex via sequence-unspecific binding to DNA (*71*, *72*). Second, Cp190 forms dimers (*73*, *74*), with each molecule capable of interacting with its own sequence-specific DNA binding partner, e.g., Su(Hw) and CTCF or CTCF and Ibf. This, in turn, would allow for Cp190-dependent cooperative binding of the whole complex to DNA. Third, Cp190 was reported to interact with Nurf301, the core component of the Nucleosome Remodeling Factor (NURF) chromatin remodeling complex (*75*). The NURF complex slides nucleosomes, which may enable Cp190-associated proteins to bind their cognate sequence motifs more efficiently. Additional experiments will be required to discriminate between these possibilities.

It has been widely assumed that *Drosophila* insulator elements restrict chromatin contacts by interacting with each other and forming chromatin loops. This model is appealing, as it would explain several phenomena observed in transgenic experiments, for example, the insulator bypass (*28*, *29*) or the long-distance regulatory interactions mediated by certain insulator elements (*21*, *76*, *77*). The results of looping tests with our Hi-C or published Micro-C (*33*) data do not support the model. While several transgenic insulators can be “bypassed” when paired with themselves, pairs of different insulators are usually not bypassed, even when both insulators bind the same bridging protein, e.g., Cp190 (*40*). This may suggest that only a specific combination of insulators can interact and form loops. Alternatively, the “bypass” may require a pair of insulators with matching ability to impair contact crossing. The former interpretation, not accounted for in our looping tests, is difficult to reconcile with expression changes caused by deletions of individual insulator from homeotic gene clusters. An insulation mechanism based on pairing between specific insulator elements implies that changes caused by the deletion of one insulator of a pair should be recapitulated, at least partially, by the deletion of the second insulator of the pair. On the contrary, deletions of individual insulator elements within homeotic gene clusters affect transcriptional regulation by distinct regulatory elements and lead to unique homeotic phenotypes (*17*, *19*, *50*).

*homie* and *nhomie* are the insulator elements that flank the *even skipped* (*eve*) locus. The transgenes containing these elements can participate in trans-regulatory interactions over distances greater than 1 Mb. Separated by ~17 kb in their endogenous location, the two form a chromatin loop detected by Micro-C (*33*). *homie* requires Cp190 to block enhancer-promoter communications. However, the Cp190 protein is dispensable for its long-range interactions (*67*). This argues that the insulator and long-range pairing activities of this element are functionally separable. While we cannot exclude that certain insulator elements restrict chromatin contacts by interacting with each other and forming chromatin loops, this is unlikely to constitute a general mechanism of *Drosophila* insulator action. Our conclusion agrees with the observations of Batut and coauthors who found only 18 chromatin loops, which coincide with a topological boundary, from a total of 331 loops detected by Micro-C in the fly embryo (*33*).

Using the Hi-C Computational Unbiased Peak Search (HiCCUPS) algorithm (*78*), Chathoth and coauthors (*69*) reported several hundred loops formed by regions cobound by Cp190, BEAF-32, and Chromator in cultured *Drosophila* cells. These sites are predominantly the TSS of active genes. Most of these sites were not classified as insulator elements in our screen because their impact on chromatin contact crossing does not require Cp190 or CTCF. We cannot exclude that some active TSSs contain elements that impair chromatin contacts by forming loops. Additional experiments would be needed to uncouple this effect from a generic interaction between transcriptionally active genes. To conclude, the extended catalog of insulator elements uncovered in our study will provide an important resource to study the regulation of specific *Drosophila* genes and the general mechanisms that shape the folding of eukaryotic genomes.

## MATERIALS AND METHODS

### Derivation and culture of Cp190-KO and CTCF-KO cell lines

The *CTCF^y+1^* and *Cp190^3^* fly strains (*42*, *43*) were used to derive the corresponding mutant cell lines following the procedure of Simcox *et al.* (*41*) with modifications described in (*45*). Cells were cultured at 25°C in Schneider’s media (Lonza), supplemented with 10% heat-inactivated fetal bovine serum (Sigma-Aldrich), streptomycin (0.1 mg/ml), and penicillin (100 units/ml) (Gibco) under sterile conditions.

### Hi-C, library preparation, and sequencing

Hi-C was performed as described in (*9*). Briefly, 2 × 10^7^ cells were cross-linked by incubating in fix buffer [2% formaldehyde, 15 mM Hepes (pH 7.6), 60 mM KCl, 15 mM NaCl, 4 mM MgCl_2_, 0.1% Triton X-100, 0.5 mM dithiothreitol, and protease inhibitor cocktail (Roche)] for a total of 10 min at 25°C and 750 rpm on a shaker. After quenching with 5 ml of 2 M glycine, permeabilized cells were collected by centrifugation for 5 min at 4500*g* and 4°C and then washed once with 5 ml of fix buffer (without formaldehyde) and once with 1.25× NEB3 buffer (New England Biolabs), with centrifugation for 5 min at 4500*g* and 4°C each time. Permeabilized cells were resuspended in 300 μl of 1.25× Dpn II buffer (New England Biolabs) and 0.3% SDS and incubated for 1 hour at 37°C and 1000 rpm on a shaker. Triton X-100 was added to a final concentration of 2%, and the permeabilized cells were incubated for a further 1 hour at 37°C and 1000 rpm before overnight treatment with 1500 U of Dpn II (New England Biolabs) at 37°C and 1000 rpm. The restriction enzyme was inactivated by incubation for 20 min at 65°C and 1000 rpm with SDS at a final concentration of 1.3% before dilution of the lysate in 10 ml of 1× T4 DNA ligase buffer (New England Biolabs) and 1% Triton X-100 and incubation for 1 hour at 37°C and 750 rpm. The released chromatin was ligated for 4 hours at 25°C and 750 rpm with 40,000 U of T4 DNA ligase (New England Biolabs). Then, cross-links were reversed overnight at 65°C and 750 rpm in the presence of proteinase K (150 μg/ml). The resulting 3C DNA was purified by 1 hour of treatment with ribonuclease A (40 μg/ml) at 37°C and 750 rpm, phenol extraction, phenol/chloroform extraction, and ethanol precipitation. The DNA was quantified with the Qubit dsDNA (double-stranded DNA) assay (Invitrogen). Five-microgram aliquots of 3C DNA were sonicated with a Bioruptor (Diagenode) in 50-μl volumes in sonication buffer [50 mM tris-HCl (pH 8), 10 mM EDTA, and 1% SDS] to obtain a fragment range between 500 and 1500 bp. The sonicated 3C DNA was then purified by phenol/chloroform extraction and ethanol precipitation and quantified with the Qubit dsDNA assay (Invitrogen). Libraries for paired-end sequencing were made from 500-ng aliquots of sonicated 3C DNA using Illumina reagents and protocols, with size selection for products of ~800 bp. The libraries were sequenced on a HiSeq 2000 instrument (Illumina), following the manufacturer’s protocol.

### ChIP library preparation and sequencing

ChIP and qPCR analysis were performed as described in (*45*) except that chromatin was sonicated in 4 ml of 10 mM tris-HCl (pH 8.0) and 1 mM EDTA (pH 8.0) with a Branson D450 sonicator for 45 min (45 cycles of 20 s on to 40 s off; amplitude, 40%) and adjusted to 5 ml in radioimmunoprecipitation assay buffer [10 mM tris-HCl (pH 8.0), 1 mM EDTA (pH 8.0), 1% Triton X-100, 0.1% SDS, 0.1% deoxycholate, and 0.14 mM NaCl]. Isolated ChIP DNA was resuspended in 40 μl of deoxyribonuclease (DNase)–free water and used for ChIP-seq library preparation. Four microliters of precipitated DNA was diluted 10-fold and used for qPCR analysis to check the specificity of ChIP reactions. The antibodies used are listed in table S4, and the nucleotide sequences of qPCR primers are listed in table S5. For ChIP-seq library preparation, 2 ng of immunoprecipitated DNA was processed using the NEBNext Ultra II DNA Library preparation Kit for Illumina (catalog number E7645) and index oligonucleotides from NEBNext Multiplex Oligos for Illumina (catalog number E7335). Fragments of average size of 180 bp were selected with the SPRIselect Reagent Kit (Beckman Coulter Inc., #B23317), amplified for 15 cycles, pooled, and sequenced (10 libraries per one flow cell) at the Science for Life Laboratory (the national sequencing facility, Stockholm branch) with a HiSeq X instrument (HiSeq Control Software 2.2.58/RTA 1.18.64) and 1 × 51 setup using “HiSeq SBS Kit v4” chemistry. The Bcl to FastQ conversion was performed using None from the CASAVA software suite. The sequence read quality was reported in Sanger/phred33/Illumina 1.8+ scale.

### Strand-specific total RNA library preparation and sequencing

Total RNA was isolated from cultured cells using TRI Reagent according to the manufacturer’s instructions (Sigma-Aldrich, #T9424). Two hundred nanograms of total RNA was used for indexed library preparation using Ovation RNA-Seq Systems 1–16 for Model Organisms Kit (#0350 DROSOPHILA, Nugen Technology). Briefly, total RNA was treated with DNase I and reverse-transcribed using random oligonucleotide primers. The resulting complementary DNA (cDNA) was fragmented to 200 bp with a Covaris E220 focused ultrasonicator using microtubes (AFA Fiber Crimp Cap), and the cDNA fragments were end-repaired, ligated to oligonucleotide adapters, strand-separated, and subjected to 18 cycles of PCR amplification. After purification, the libraries were pooled and sequenced at the Science for Life Laboratory (the national sequencing facility, Stockholm branch) using one flow cell of HiSeq2500 (HiSeq Control Software 2.2.58/RTA 1.18.64) with a 2 × 126 setup and HiSeq SBS Kit v4 chemistry. The Bcl to FastQ conversion was performed using None from the CASAVA software suite. The sequence read quality was reported in Sanger/phred33/Illumina 1.8+ scale.

### Hi-C analysis

#### Primary data processing and normalization

Sequence read mapping to the *D. melanogaster dm3* genome release (see statistics in table S1), filtering, and normalization were performed as previously described (*9*). The resulting statistics on the number of observed contacts for each pair of restriction fragments and the number of expected contacts from a low-level probabilistic model, which considers local GC content and the Dpn II restriction fragment length (*79*), is reported in table S2. Technically corrected contact matrices were generated by calculating ratios between the total observed reads and the expected reads based on the above model. Bin-contact pair maps were transformed to the gcmap format using gcMapExplorer bc2cmap with iterative correction (IC) gcMapExplorer normIC (*80*).

For pairwise comparisons of Hi-C contact matrices, Spearman’s rank correlation (ρ) and Pearson moment correlation (*r*) coefficients were calculated using the cor function in R (www.R-project.org/). To avoid spurious experimental noise, the matrices were filtered to remove bins with observed contacts of less than two, and contacts within individual bins (the diagonal of the contact matrix) and between bins separated by more than 1.6 Mb were not considered. The similarity between Hi-C contact matrices of individual experiments was evaluated by hierarchical clustering as implemented in hclust function with distances between experiments calculated as 1 − absolute value of correlation coefficient. The cluster stability was evaluated by performing four variations of the procedure, i.e., using ρ or *r* and two agglomeration methods: complete and average Unweighted Pair Group Method with Arithmetic mean (UPGMA). For bins equal or larger than 40 kb, the clustering was robust to variations (Fig. 1C and fig. S4).

All comparisons between Hi-C, ChIP-seq, and RNA-seq datasets were performed using datasets in *dm3* genomic coordinates. In addition, for visualization and reporting purposes, the positions of insulator regions and contact heatmaps were also transposed to the *dm6* genome release coordinates using the UCSC LiftOver tool.

#### Data visualization

Contact matrices for replicate experiments at a 5-kb bin resolution were combined, converted to gcmap format, subjected to IC correction, and displayed using gcMapExplorer browser (*80*).

#### Distance-scaling factor (γ) computation and assignment to specific regions

The distance-scaling factor (γ) was computed for each Dpn II restriction fragment as described in (*79*). TAD borders were called from restriction fragment–level contact matrices of individual Hi-C experiments with control cells using the 95th percentile for γ genome-wide as the threshold (*9*). The accuracy of TAD border positions was estimated by comparing the two replicate experiments (fig. S6) and was set to 2000 bp. That is, TAD borders identified in replicate experiments at a distance of 2000 bp or less were considered identical. To assign γ values to insulator protein bound regions, coordinates of each region were compared to the coordinates of the Dpn II restriction fragments and the overlapping fragments selected for further analysis. Insulator protein bound regions were segmented into 200-bp sliding windows, and the coordinates of these windows were compared to the coordinates of the selected Dpn II restriction fragments. Bedtools intersect -wa -wb was used for all coordinate comparisons (*81*). From this, the weighted γ of each 200-bp sliding window was calculated from the γ of individual Dpn II restriction fragments, taking into account the degree to which the window overlapped these fragments. The highest value of all windows contained within an insulator protein bound region was taken to represent the region’s γ.

#### Definition of classes of insulator proteinbound regions with systematic increase in chromatin contact crossing after Cp190-KO or CTCF-KO and calculation of FDRs

The systematic increase in contact crossing was defined as a negative shift in Δγ values, which was unlikely to happen by chance given the null distribution approximated by Δγ at 344 random sites not bound by any of the insulator protein profiled. To this end, the proportion of sites with the bottom 10% of Δγ at a class of insulator protein regions was compared to that at the random sites using one-sided Fisher’s exact test. For classes of insulator protein regions that displayed systematic chromatin contact crossing upon Cp190-KO or CTCF-KO, Δγ values for the bottom 5th, 10th, and 15th percentiles among 344 random sites were used as corresponding FDR thresholds to call the regions significantly affected by knockdown (putative chromatin insulator regions).

#### Calculations of contact crossing difference curves

To account for sampling biases between Hi-C experiments, the contact matrices were further normalized using multiHiCcompare algorithm (*82*), which provides cyclic loess and fast loess methods adapted to jointly normalize the Hi-C data with more than two groups and multiple samples per group (supplementary code file: hic_normalize.R). The normalized contact matrices were used to calculate numbers of contacts between pairs of Hi-C bins around selected central bins (*b*_0_) starting from the two bins immediately adjacent to *b*_0_ and followed by pairs at progressively larger distances of up to 60 bins (300 kb = 60 × 5 kb) (supplementary code file: gen_ContactCcrossingCurves.sh). The values for the corresponding bin pairs calculated for mutant and control cells were subtracted to yield the contact crossing difference curves.

#### Insulator looping test

Putative chromatin insulator sites were grouped by chromosome and sorted by their starting position to identify the closest pairs (i.e., *i1* and *i2*, as illustrated in Fig. 6C). Each pair of the closest insulator elements (*i1* and *i2*) located at a distance *L* was supplemented with three matching control pairs. The first control pair consisted of one of the insulators (*i1*) and the control region (*c1*) located at a distance of *L*/2 toward the second insulator (*i2*). The second control pair consisted of the insulator (*i1*) and the control region (*c2*) located at the distance *L* to the left (upstream) of *i1*. Last, the third control pair included control sites *c2* and *c3*, with the latter located at distance *L* to the left of *c2*. The number of contacts between regions of each pair was calculated using HiCmapTools (supplementary code file: gen_InsulatorLoopingTest.sh) (*83*). To perform the test on Micro-C data, the contact matrices in .cool format were downloaded from the National Center for Biotechnology Information Gene Expression Omnibus (accession numbers GSM5224834 and GSM5224835), transformed into .ginteraction format using the hicConvertFormat function of Galaxy HiCExplorer 3 software suit (*84*), and used to calculate the number of contacts between paired sites as described above.

### ChIP-seq analysis

Reads were aligned to the *D. melanogaster dm3* genome assembly using bowtie2 (*85*) and the following parameters: --phred33 -p 8. Reads with MAPQ scores less than 30 were removed using samtools (*86*) view command and -q 30. Genomic read count profiles were computed from the filtered bowtie2 alignments using pyicos convert software (*87*) and the following parameters: -f sam -F bed_wig -x 180 -O. The resulting read count profiles were normalized to the number of corresponding MAPQ 30–filtered sequencing reads using a custom R script. Positions of ChIP-seq signal maxima within regions significantly enriched in both replicate experiments with the chromatin from control cells were identified with MACS2 (v2.1.2) (*56*) callpeak command using the following parameters: -f SAM -g dm --keep-dup all --fe-cutoff 8. The resulting bed files were extended ±300 bp. Genomic positions of the regions above defined for each insulator-binding protein were compared pairwise in order Mod(mdg4), Cp190, Ibf1, CTCF, and Su(Hw). Thus, regions enriched by ChIP with antibodies against Mod(mdg4) were checked for overlap with regions enriched with antibodies against Cp190. The resulting three groups, i.e., bound by both Mod(mdg4) and Cp190, bound by just Mod(mdg4), or bound by just Cp190, were further compared to regions enriched by ChIP with antibodies against Ibf and so on. The resulting cobinding classes were designated with combinations of single letters representing individual insulator proteins bound.

To calculate ChIP-seq signal scores, read count profiles of individual insulator protein bound regions or TSSs were extracted from the normalized genomic read count profiles of corresponding proteins using BEDTools (*81*) intersect function with parameters -wa -wb. The region-specific profiles were then used to calculate the average read count per base pair ChIP-seq signal scores. TSS regions were defined by a ±1000-bp extension of TSS positions obtained from UCSC Genome Browser (*88*).

### RNA-seq analysis

Paired-end reads were mapped to the *D. melanogaster dm3* genome assembly using STAR_v2.6.1a (*89*) with default parameters. Unmapped or nonunique mapped reads were discarded (see statistics in table S6). The gene transcription value was quantified as read count with --quantMode GeneCounts. Genes with transcription values within the top quartile were designated as transcriptionally active (TSS-high). Conversely, genes with transcription values within the bottom quartile were designated as transcriptionally inactive (TSS-low). We identified differentially transcribed genes using DESeq2 (*65*) with log_2_ fold change = 1 and Wald significance test with *P* = 0.05 as thresholds. PCA and identification of the top 20 most variable genes were performed after regularized log transformation of transcription values. Figure S12 provides an overview of the entire data analysis pipeline.

## Supplementary Materials

### This PDF file includes:

Figs. S1 to S12 References

### Other Supplementary Material for this manuscript includes the following:

Tables S1 to S6

Supplementary code file
