# Supplementary material for: Topological screen identifies hundreds of Cp190- and CTCF-dependent Drosophila chromatin insulator elements
Source: Sci Adv. 2023 Feb 3;9(5):eade0090. doi: 10.1126/sciadv.ade0090 (PMC9897668; doi:10.1126/sciadv.ade0090)
Supplement: Supplementary file 1 — Figs. S1 to S12 References [file sciadv.ade0090_sm.pdf]

Science Advances
AAAS

Supplementary Materials for

Topological screen identifies hundreds of Cp190- and CTCF-dependent *Drosophila* chromatin insulator elements

Tatyana G. Kahn *et al.*

Corresponding author: Yuri B. Schwartz, yuri.schwartz@umu.se; Jia-Ming Chang, chang.jiaming@gmail.com

Sci. Adv. **9**, eade0090 (2023)
DOI: 10.1126/sciadv.ade0090

The PDF file includes:

Figs. S1 to S12
Legends for tables S1 to S6
Legend for supplementary code file
References

Other Supplementary Material for this manuscript includes the following:

Tables S1 to S6
Supplementary code file

Supplementary figures

Figure S1

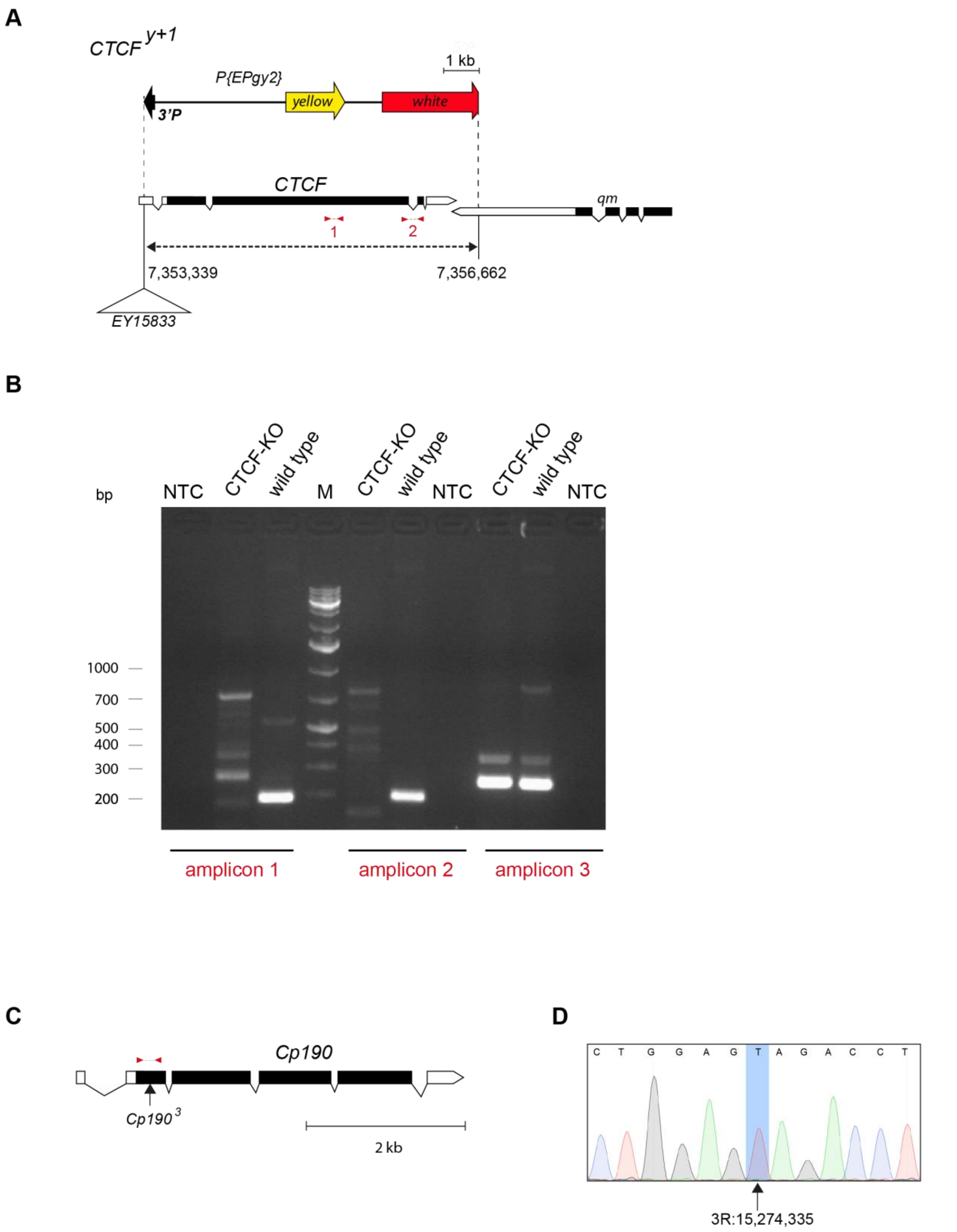

Figure S1. Characterization of CTCF 19.7-1c (CTCF-KO) and CP-R6 (Cp190-KO) cultured cell lines. A. Schematic representation of *CTCF* $^{y+1}$ allele. The allele corresponds to

3.3kb deletion (dashed line) that removes most of the *CTCF* open reading frame and a small part of the 3' UTR of the adjacent *qm* gene. Exons are shown as boxes, coding parts marked with back. Coordinates of deletion breakpoints are according to *Drosophila melanogaster* genome release BDGP R6/*dm6*, 2014. The $CTCF^{y+1}$ deletion was generated by imprecise excision of $P[EPgy]CTCF^{EY15833}$ transposon (indicated as white triangle) and still contains some of its constituents including functional *yellow* gene and part of the *white* gene (shown above the molecular map of the *CTCF* locus). Note, that the schematic of $P[EPgy]CTCF^{EY15833}$ remnants is in different scale than that of the *CTCF* map. Positions of amplicons used to confirm the $CTCF^{y+1}$ deletion in CTCF-KO cell lines are indicated with red lines. **B**. Genomic DNAs from CTCF 19.7-1c (CTCF-KO) and Ras 3 (wild type) cells were used for PCR with CTCFfw4 and CTCFrev3 primers (amplicon 1, expected product size - 189bp), CTCFfw5 and CTCFrev2 (amplicon 2, expected product size - 196bp) as well as BP3 and BP4 (amplicon 3, positive control outside the *CTCF* locus, expected product size – 258bp). As shown by electrophoresis of PCR products in 2% agarose gel, only reactions with genomic DNA from the wild-type cells yield single products corresponding to amplicons 1 and 2. This confirms that all CTCF-KO cells lack *CTCF* open reading frame. **C.** Molecular map of *Cp190* gene. Arrow indicates the position of C to T transition (Q61Stop) in the $Cp190^{3}$ allele. Red line indicates the location of the PCR product sequenced to genotype Cp190-KO cells. **D.** Genotyping of CP-R6 (Cp190-KO) cells. Genomic DNA from CP-R6 cells was amplified by PCR with CP190fw and CP190-Ncolrev primers, and the product of expected size – 382bp sequenced. The fragment of Sanger sequencing chromatogram illustrates that all CP-R6 chromosomes contain T instead C (blue shaded rectangle) at position 3R:15,274,335 (genome release BDGP R6/*dm6*, 2014).

Figure S2

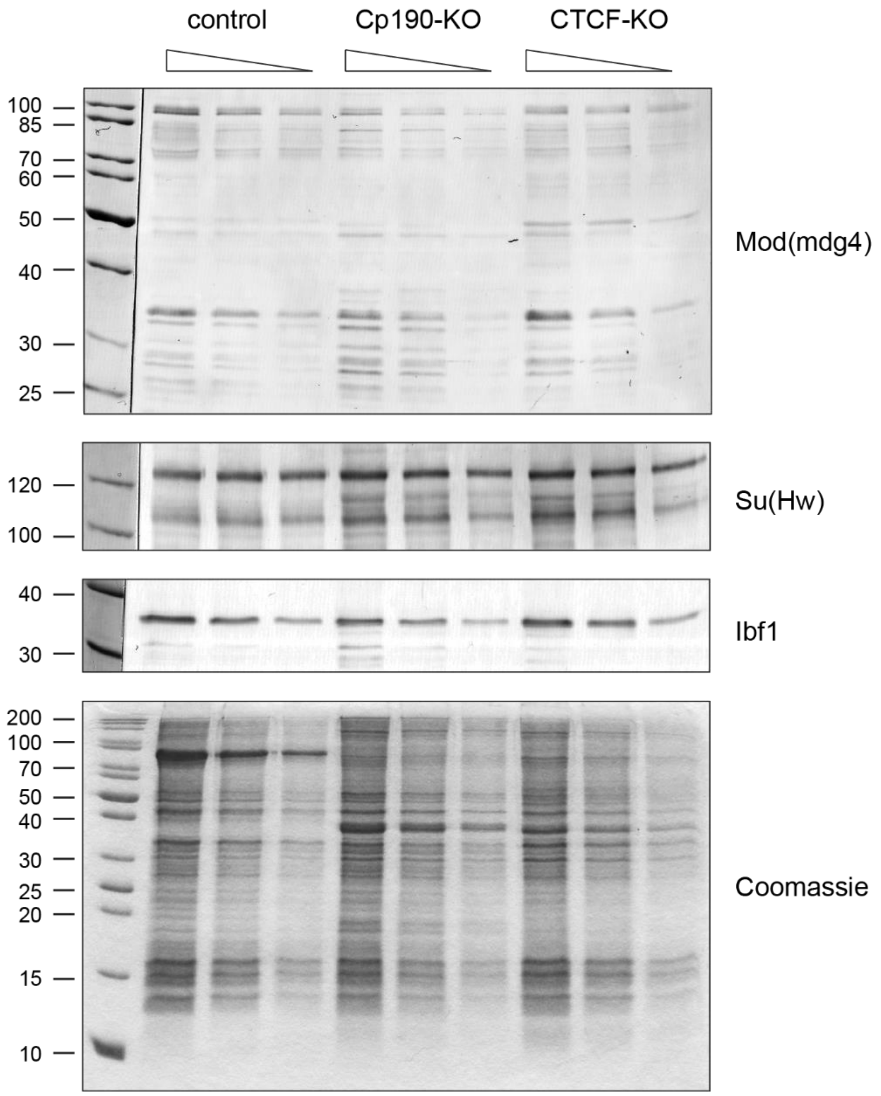

Figure S2. Ablation of Cp190 and CTCF does not affect the overall levels of other insulator proteins. Two-fold dilutions of total nuclear protein from control (Ras3), Cp190-KO (CP-R6) and CTCF-KO (CTCF 19.7-1c) cells were analysed by western-blot with antibodies against Mod(mdg4), Su(Hw) and Ibf1. Coomassie stained gel of corresponding total nuclear protein samples and western-blot with antibodies against Pc (Figure 1A) were used to control equal loading. Positions of molecular weight markers (in kDa) are indicated to the left.

Figure S3

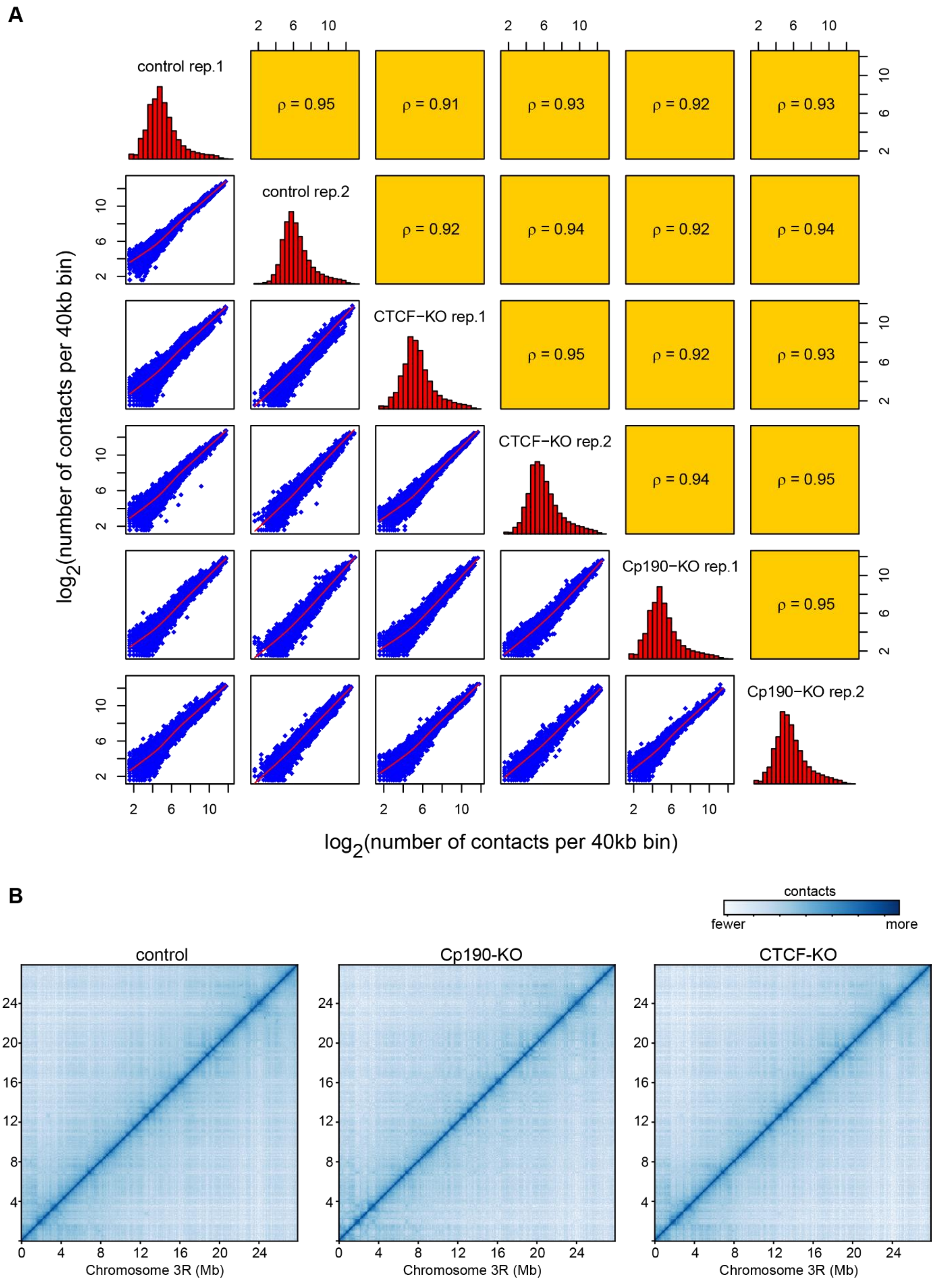

Figure S3. Overall chromatin contacts in the control, Cp190-OK and CTCF-KO cells are similar. A. Pairwise correlations between numbers of contacts for 40kb genomic

segments (Hi-C bins) measured in individual experiments. Histograms along the diagonal show distributions of contact numbers for each experiment. Scatter plots below the diagonal illustrate the correspondence between contact numbers for individual bins (blue dots). Orange squares above the diagonal show corresponding Spearman's rank correlation coefficients. To avoid spurious experimental noise, contacts within individual bins (the diagonal of contact matrix) as well as contacts between bins separated by more than 1.6Mb were not considered. **B.** Chromatin contacts over the right arm of *Drosophila melanogaster* chromosome 3 were counted at 40kb resolution, normalized by iterative correction and plotted using *gcMapExplorer* software. To allow simultaneous visualization of the low- and high-frequency contacts, the heat map representation was scaled as logarithm of the map.

Figure S4

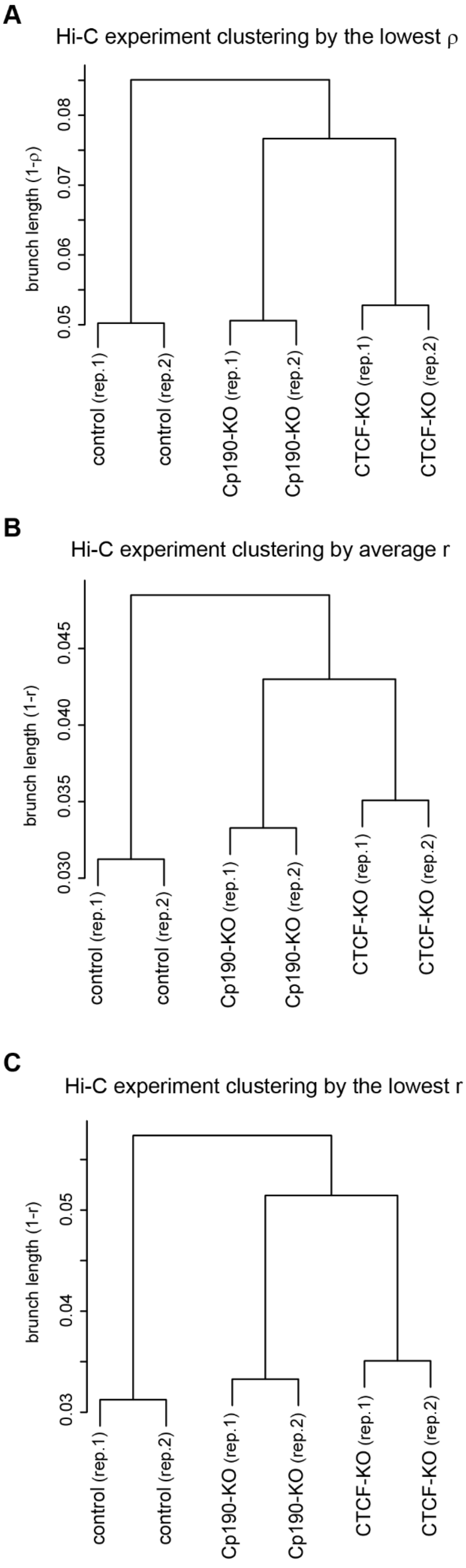
A
Hi-C experiment clustering by the lowest ρ
brunch length (1-ρ)
0.05
0.06
0.07
0.08
control (rep.1)
control (rep.2)
Cp190-KO (rep.1)
Cp190-KO (rep.2)
CTCF-KO (rep.1)
CTCF-KO (rep.2)
B
Hi-C experiment clustering by average r
brunch length (1-r)
0.030
0.035
0.040
0.045
control (rep.1)
control (rep.2)
Cp190-KO (rep.1)
Cp190-KO (rep.2)
CTCF-KO (rep.1)
CTCF-KO (rep.2)
C
Hi-C experiment clustering by the lowest r
brunch length (1-r)
0.03
0.04
0.05
control (rep.1)
control (rep.2)
Cp190-KO (rep.1)
Cp190-KO (rep.2)
CTCF-KO (rep.1)
CTCF-KO (rep.2)

Figure S4. For bins equal or larger than 40kb hierarchical clustering of individual Hi-C experiments is robust to parameter changes. Chromatin contacts measured in individual Hi-C experiments were assigned to 40kb genomic segments (Hi-C bins) and clustered based on the lowest Spearman's rank correlation coefficient for the group (**A**), average Pearson correlation coefficients for the group (**B**) or the lowest Pearson correlation coefficients for the group (**C**). The resulting dendrograms (also see Figure 1C) are the same regardless of the approach. Datasets assigned to larger (80kb or 160kb) bins yield equivalent dendrograms, which are also robust to parameter changes. When chromatin contacts are binned to smaller segments (5kb, 10kb and 20kb), the clustering outcomes start to vary depending on the kind of correlation coefficient (Spearman's rank or Pearson) and the grouping approach (lowest or average for the group) is used. We, therefore, consider the latter results unreliable.

Figure S5

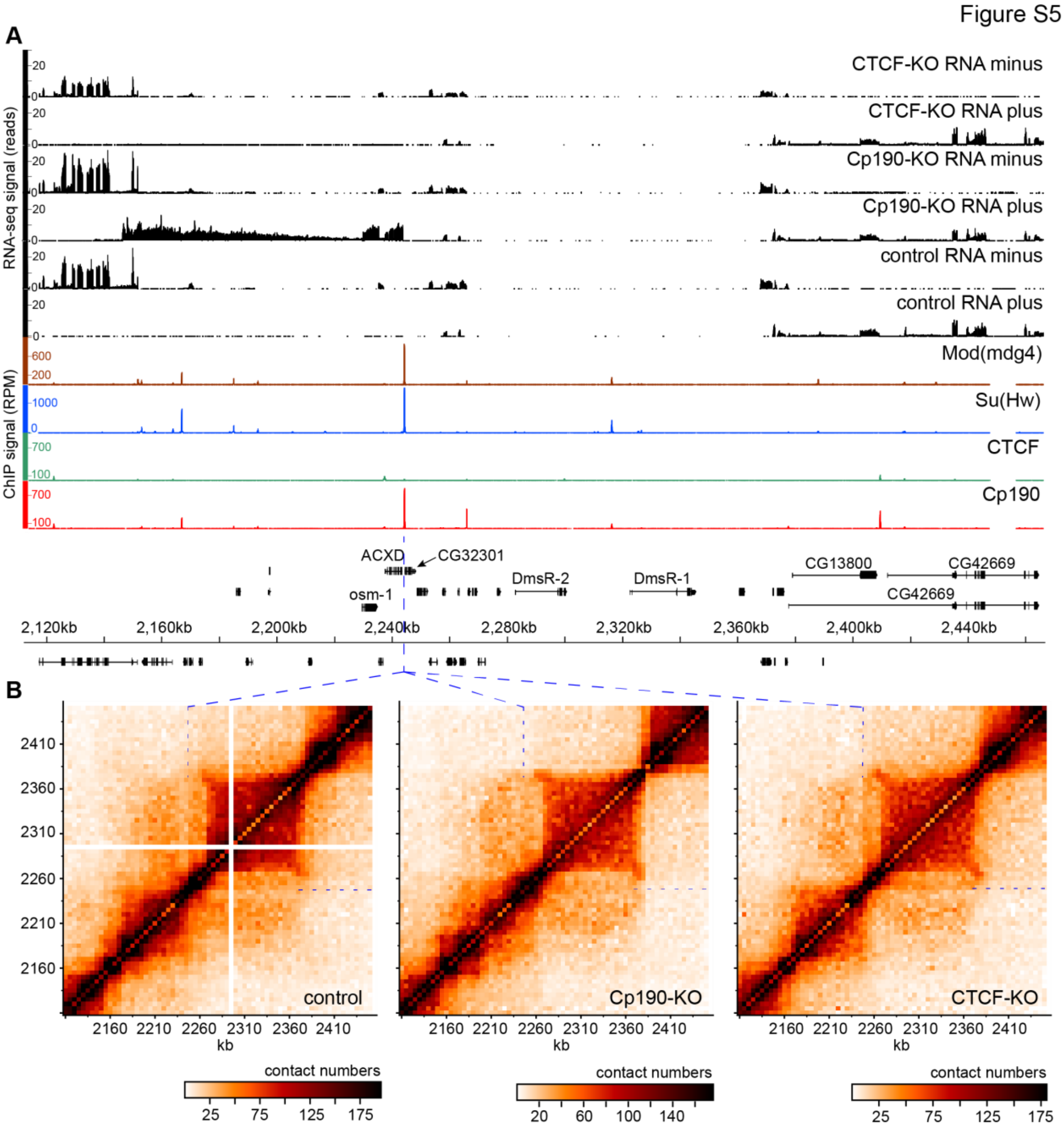

Figure S5. Chromatin topology around the *62D* insulator element. A. Genomic organization of the 62D region. ChIP-seq profiles for Cp190, CTCF, Su(Hw) and Mod(mdg4) proteins in control cells are displayed as number of sequencing reads per position per million of total reads. RNA-seq profiles from control, Cp190-KO and CTCF-KO cells are displayed separately for each DNA strand as number of sequencing reads per position. Positions and the exon structure of annotated transcripts are shown above (transcribed from left to right) or below (transcribed from right to left) the scale in *dm6*, 2014 genome release coordinates. The position of the *62D* insulator element is indicated with blue dashed lines. **B.** Chromatin contacts within the 62D region in control, Cp190-KO and CTCF-KO cells. Contacts measured by individual Hi-C experiments were assigned to 5kb bins and

normalized by iterative correction. The data from replicate experiments were combined and visualized with the *gcMapExplorer* software.

Figure S6

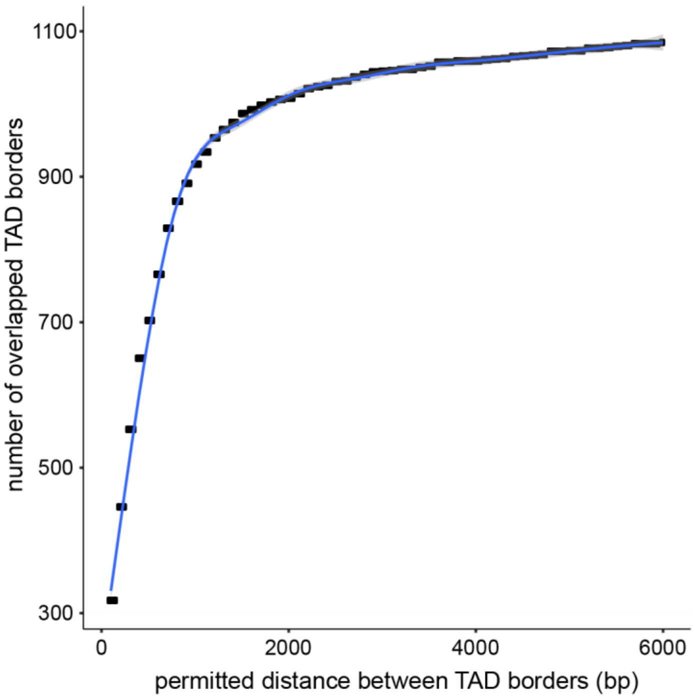

Figure S6. The optimal accuracy of TAD border mapping. To estimate the accuracy of TAD border positions, the number of overlapped TAD borders defined in two replicate experiments in the control cells was plotted against the permitted distance between two borders considered overlapped. From this, TAD borders identified in replicate experiments at a distance of 2000bp or less were considered identical.

Figure S7

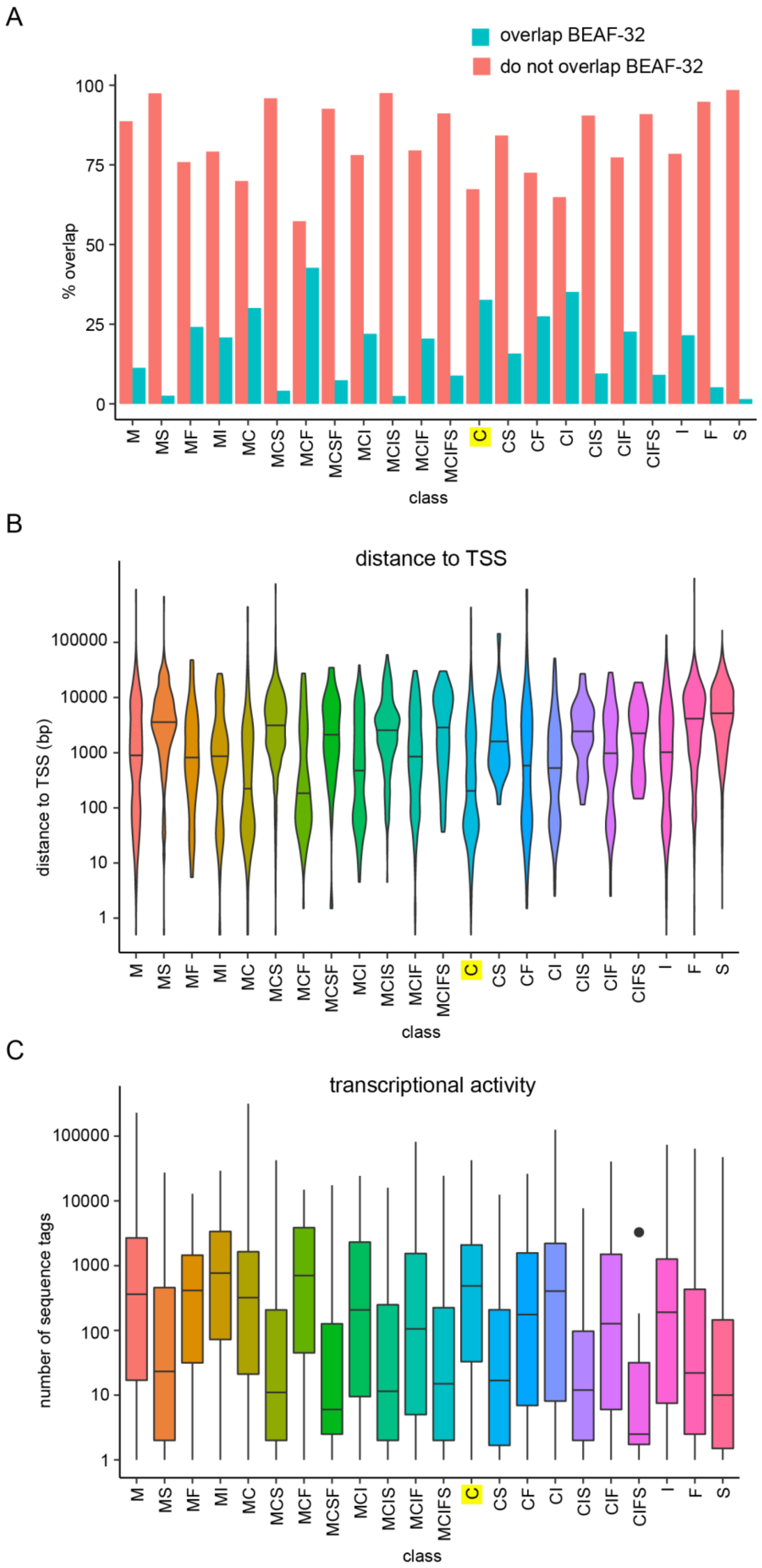
A
overlap BEAF-32
do not overlap BEAF-32
% overlap
100
75
50
25
0
M MS MF MI MC MCS MCF MCSF MCI MCIS MCIF MCIFS C CS CF CI CIS CIF CIFS I F S
class
B
distance to TSS
distance to TSS (bp)
100000
10000
1000
100
10
1
M MS MF MI MC MCS MCF MCSF MCI MCIS MCIF MCIFS C CS CF CI CIS CIF CIFS I F S
class
C
transcriptional activity
number of sequence tags
100000
10000
1000
100
10
1
M MS MF MI MC MCS MCF MCSF MCI MCIS MCIF MCIFS C CS CF CI CIS CIF CIFS I F S
class

Figure S7. Relation between classes of insulator protein bound regions, proximity to transcription start sites, transcription and BEAF-32. A. Bar-plots indicate fraction of insulator protein bound regions of different classes that either overlap or do not overlap with BEAF-32 bound regions mapped by modENCODE (*38*). Here and in **B** and **C**, the yellow shading marks the C class discussed in the text. **B.** Violin-plots show distances to the closest TSS for different classes of insulator protein bound regions. Horizontal lines indicate medians. **C.** Transcriptional activity of the gene with the closest TSS for different classes of insulator protein bound regions. Box-plots indicate the median and span interquartile range with whiskers extending 1.5 times the range and outliers shown as black dots.

Figure S8

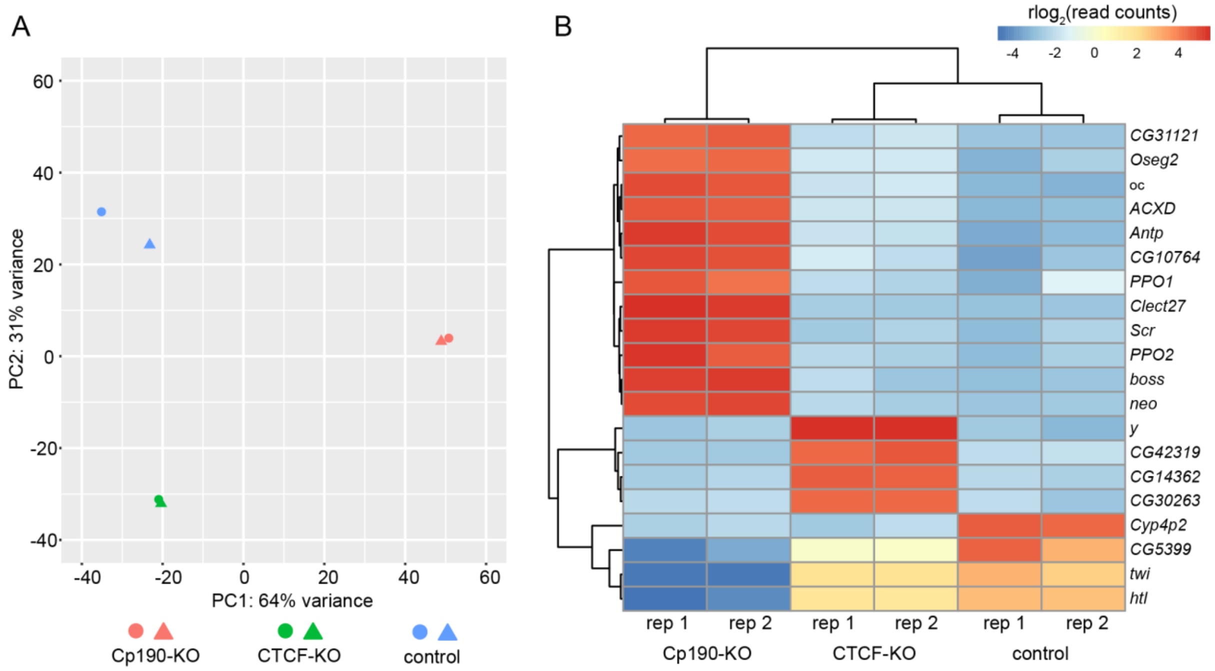

Figure S8. Differences in transcription between Cp190-KO, CTCF-KO and control cells. A. Principal component analysis of replicate RNA-seq experiments in the three cell lines. **B.** Clustered heatmap of RNA-seq signals for the twenty most differentially transcribed genes. Both analyses indicate that experiments are highly reproducible and that Cp190-KO is the most different of the three cell lines.

Figure S9

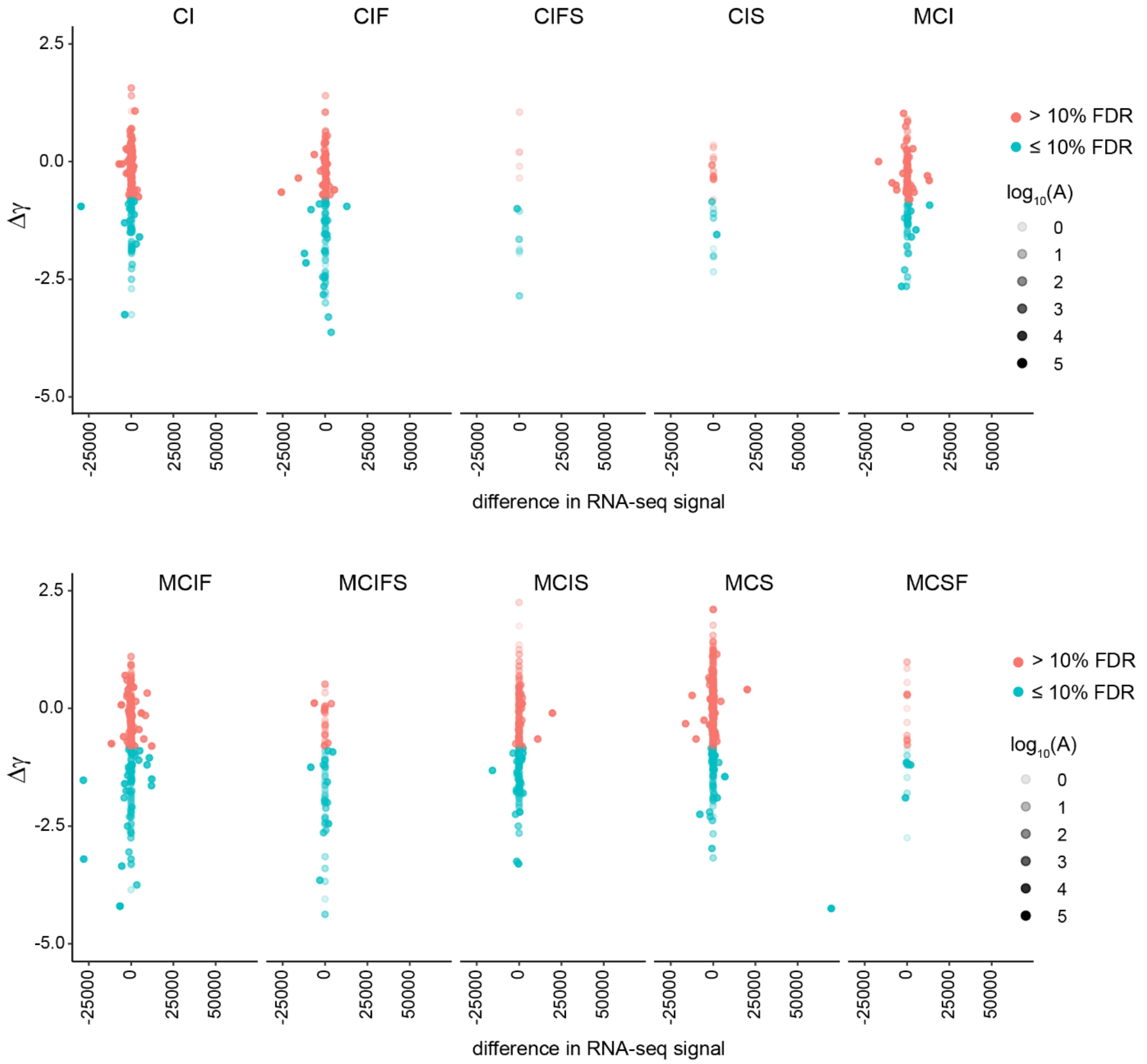

Figure S9. No correlation between changes in distance-scaling factors (Δγ) and transcription of the nearby genes. Scatter-plots compare the changes in distance-scaling factors (Δγ ≤ 10% FDR coloured in cyan; Δγ > 10% FDR coloured in pink) at insulator protein binding sites of various classes to differences in RNA-seq signals of genes closest to these sites after Cp190 knock-out. The average RNA-seq signals between control and Cp190-KO cells (A) in log10 scale are indicated by variable point intensities.

Figure S10

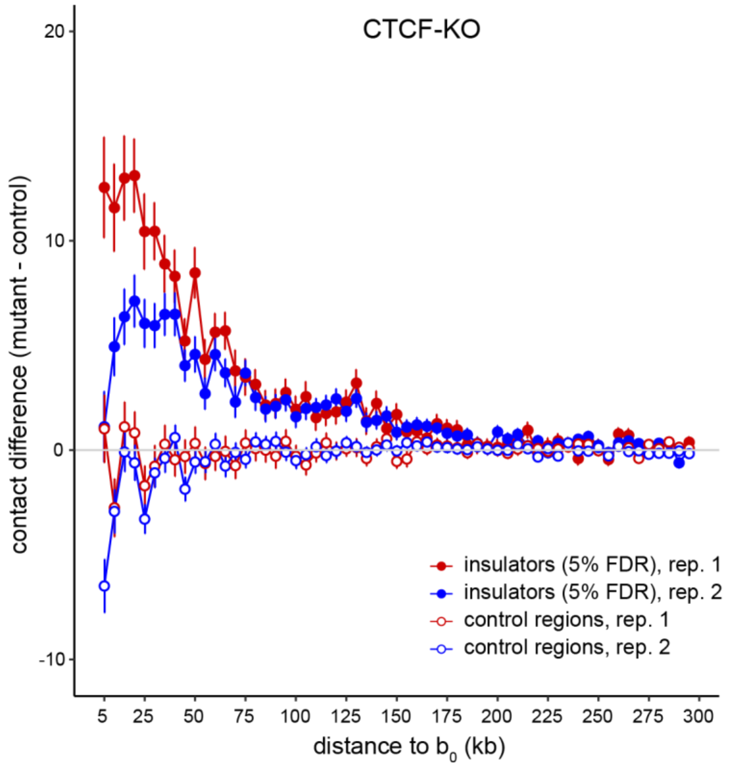

Figure S10. Action range of CTCF-dependent insulators. Average contact crossing difference curves for CTCF-dependent insulator elements (filled circles) and randomly selected control regions that do not bind any insulator proteins (empty circles) determined from two replicate Hi-C experiments (indicated with red and blue colours). Note that at close distances (5-10kb) the estimate of chromatin contact frequency from proximity ligation (the underlying principle of Hi-C method) becomes less reliable because, most of the time, chromatin fragments are sufficiently close to each other and successful ligation is largely dependent on random chance.

Figure S11

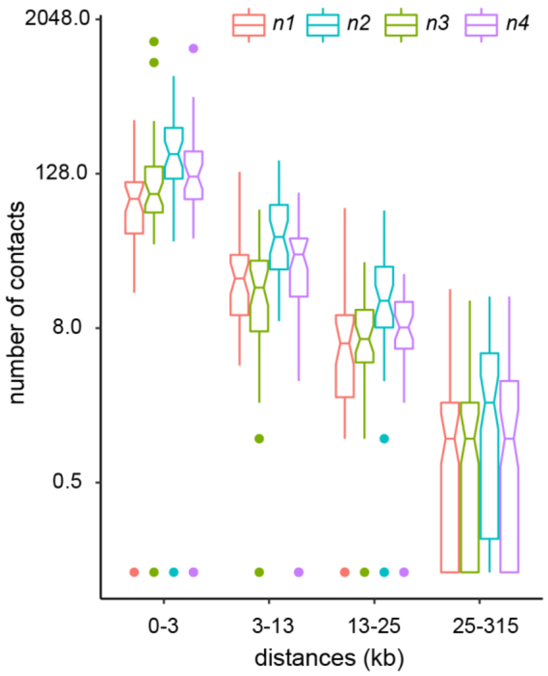

Figure S11. Looping test with high-resolution Micro-C data. Pairs of the closest insulator elements (defined at 15% FDR) were split in groups depending on their separation in the linear genome. The number of contacts between the paired insulators (*n1*) was plotted (red box-plots) alongside the number of contacts between corresponding pairs of control regions (blue (*n2*), green (*n3*) and purple (*n4*) boxplots). Notches mark 95% confidence intervals of the medians.

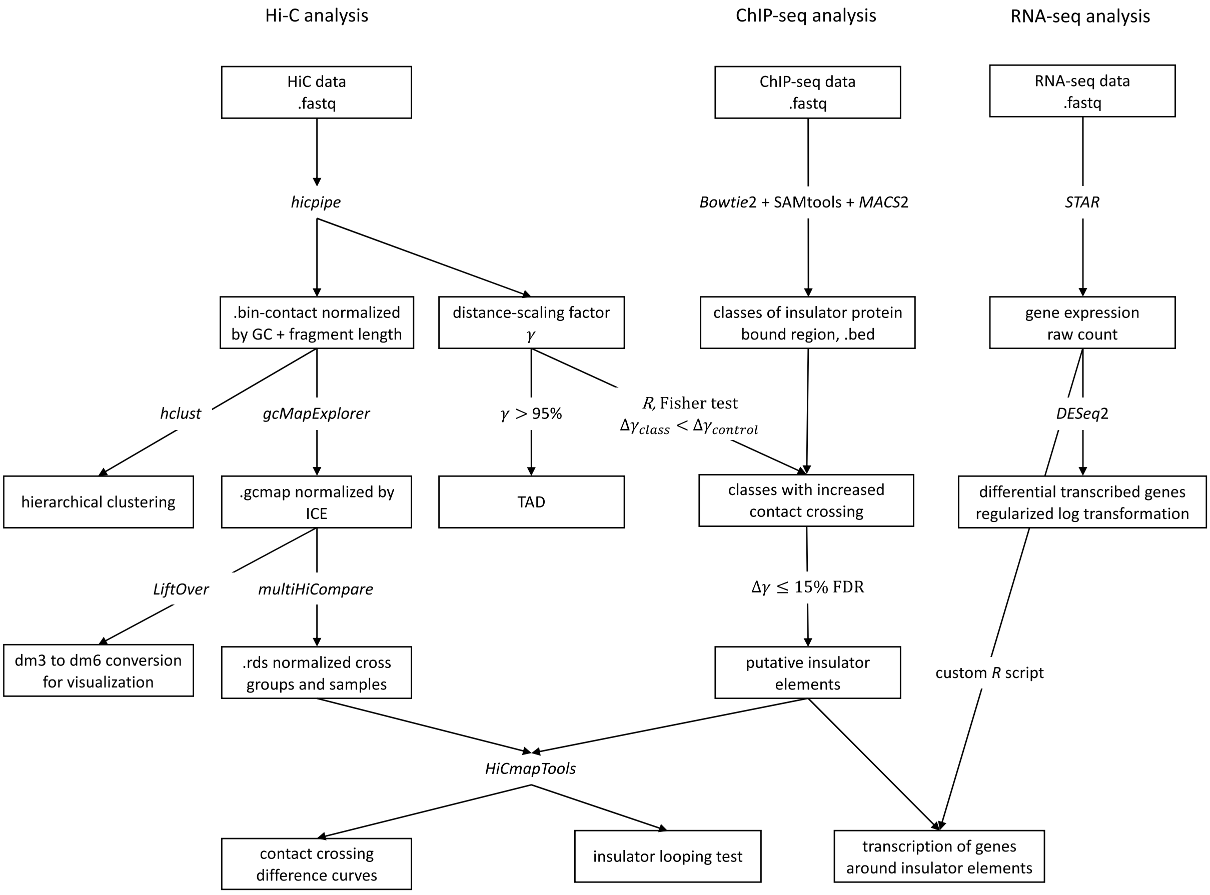

Figure S12. The diagram of the analysis pipeline. The pipeline consisted of three intersecting brunches dedicated to Hi-C, ChIP-seq, and RNA-seq analyses. **Hi-C analysis**: the raw sequencing data were processed by *hicpipe*, which returned the contact matrices normalized by GC content and fragment length as well as distance-scaling factor (γ) values. *hclust* was used for hierarchical clustering of contact matrices. For visualization, the contact matrices were normalized using the IC algorithm implemented in *gcMapExplorer normIC* and genomic coordinates converted from *dm3* to *dm6* using *LiftOver*. *multiHiCompare* was used to jointly normalize the Hi-C data across samples and conditions. **ChIP-seq analysis**: the raw sequencing data were processed by *Bowtie*2, *SMAtools*, and *MACS*2 to identify regions bound by individual insulator proteins. These were further grouped into classes based on co-binding of specific insulator protein combinations. Changes in distance-scaling factors (Δγ) within insulator protein bound regions upon Cp190-KO and CTCF-KO were assessed by Fisher exact test to identify the classes whose insulator protein bound regions displayed systematic increase in the contact crossing compared to control regions. Putative chromatin insulator elements were selected from these classes if their Δγ passed 15% FDR threshold.

For these elements, *HiCmapTools* was used to perform the looping test and derive the contact crossing difference curves. **RNA-seq analysis**: the raw sequencing data were processed using *STAR* and the results summarized as read counts per gene. Differentially transcribed genes were identified using *DESeq*2 and changes in transcription of genes around insulator elements were evaluated using the *STAR*-output read counts per gene files.

Table S1. Hi-C mapping statistics.

Table S2. Digestion statistics from *hicpipe*. See reference 79 for detailed description of the algorithm.

Table S3. The list of Cp190- and/or CTCF-dependent chromatin insulator elements. Note the FDR at which a putative insulator was called. We consider elements called at 5% FDR - high confidence, 10% FDR - moderate confidence and 15% FDR - low confidence.

Table S4. The list of antibodies used in the study.

Table S5. The list of PCR primers.

Table S6. Statistics of RNA-seq read mapping. The “number of input reads” statistic include uniquely mapped reads plus reads mapped to multiple loci plus reads mapped to too many loci plus unmapped reads. The “mapped to multiple loci” statistic describes reads mapped to more than one genomic position but to less than the limit set by the STAR parameter --*outFilterMultimapNmax*, default is 10. The “mapped to too many loci” statistic describes reads that mapped to more than the limit in the above (i.e. more than 10).

Supplementary code file. Custom *R* and *bash* scripts. These scripts were used to jointly normalize the Hi-C data with more than two groups, calculate contact crossing difference curves and perform the insulator looping test.

REFERENCES AND NOTES

1. B. Bintu, L. J. Mateo, J.H. Su, N. A. Sinnott-Armstrong, M. Parker, S. Kinrot, K. Yamaya, A. N. Boettiger, X. Zhuang, Super-resolution chromatin tracing reveals domains and cooperative interactions in single cells. *Science* **362**, (2018).

2. L. J. Mateo, S. E. Murphy, A. Hafner, I. S. Cinquini, C. A. Walker, A. N. Boettiger, Visualizing DNA folding and RNA in embryos at single-cell resolution. *Nature* **568**, 49–54 (2019).

3. H. D. Ou, S. Phan, T. J. Deerinck, A. Thor, M. H. Ellisman, C. C. O'Shea, ChromEMT: Visualizing 3D chromatin structure and compaction in interphase and mitotic cells. *Science* **357**, (2017).

4. J. H. Su, P. Zheng, S. S. Kinrot, B. Bintu, X. Zhuang, Genome-scale imaging of the 3D organization and transcriptional activity of chromatin. *Cell* **182**, 1641–1659.e26 (2020).

5. Q. Szabo, A. Donjon, I. Jerković, G. L. Papadopoulos, T. Cheutin, B. Bonev, E. P. Nora, B. G. Bruneau, F. Bantignies, G. Cavalli, Regulation of single-cell genome organization into TADs and chromatin nanodomains. *Nat. Genet.* **52**, 1151–1157 (2020).

6. Q. Szabo, D. Jost, J.M. Chang, D. I. Cattoni, G. L. Papadopoulos, B. Bonev, T. Sexton, J. Gurgo, C. Jacquier, M. Nollmann, F. Bantignies, G. Cavalli, TADs are 3D structural units of higher-order chromosome organization in Drosophila. *Sci. Adv.* **4**, eaar8082 (2018).

7. J. R. Dixon, S. Selvaraj, F. Yue, A. Kim, Y. Li, Y. Shen, M. Hu, J. S. Liu, B. Ren, Topological domains in mammalian genomes identified by analysis of chromatin interactions. *Nature* **485**, 376–380 (2012).

8. E. P. Nora, B. R. Lajoie, E. G. Schulz, L. Giorgetti, I. Okamoto, N. Servant, T. Piolot, N. L. van Berkum, J. Meisig, J. Sedat, J. Gribnau, E. Barillot, N. Blüthgen, J. Dekker, E. Heard, Spatial partitioning of the regulatory landscape of the X-inactivation centre. *Nature* **485**, 381–385 (2012).

9. T. Sexton, E. Yaffe, E. Kenigsberg, F. Bantignies, B. Leblanc, M. Hoichman, H. Parrinello, A. Tanay, G. Cavalli, Three-dimensional folding and functional organization principles of the Drosophila genome. *Cell* **148**, 458–472 (2012).

10. L. A. Mirny, M. Imakaev, N. Abdennur, Two major mechanisms of chromosome organization. *Curr. Opin. Cell Biol.* **58**, 142–152 (2019).

11. Q. Szabo, F. Bantignies, G. Cavalli, Principles of genome folding into topologically associating domains. *Sci. Adv.* **5**, eaaw1668 (2019).

12. E. J. Banigan, L. A. Mirny, Loop extrusion: Theory meets single-molecule experiments. *Curr. Opin. Cell Biol.* **64**, 124–138 (2020).

13. O. L. Kantidze, S. V. Razin, Weak interactions in higher-order chromatin organization. *Nucleic Acids Res.* **48**, 4614–4626 (2020).

14. P. K. Geyer, V. G. Corces, DNA position-specific repression of transcription by a Drosophila zinc finger protein. *Genes Dev.* **6**, 1865–1873 (1992).

15. C. Holdridge, D. Dorsett, Repression of hsp70 heat shock gene transcription by the suppressor of hairy-wing protein of Drosophila melanogaster. *Mol. Cell. Biol.* **11**, 1894–1900 (1991).

16. A. Udvardy, E. Maine, P. Schedl, The 87A7 chromomere. Identification of novel chromatin structures flanking the heat shock locus that may define the boundaries of higher order domains. *J. Mol. Biol.* **185**, 341–358 (1985).

17. S. Barges, J. Mihaly, M. Galloni, K. Hagstrom, M. Muller, G. Shanower, P. Schedl, H. Gyurkovics, F. Karch, The Fab-8 boundary defines the distal limit of the bithorax complex iab-7 domain and insulates iab-7 from initiation elements and a PRE in the adjacent iab-8 domain. *Development* **127**, 779–790 (2000).

18. A. C. Bell, G. Felsenfeld, Methylation of a CTCF-dependent boundary controls imprinted expression of the Igf2 gene. *Nature* **405**, 482–485 (2000).

19. W. Bender, M. Lucas, The border between the ultrabithorax and abdominal-A regulatory domains in the Drosophila bithorax complex. *Genetics* **193**, 1135–1147 (2013).

20. N. Engel, J. L. Thorvaldsen, M. S. Bartolomei, CTCF binding sites promote transcription initiation and prevent DNA methylation on the maternal allele at the imprinted H19/Igf2 locus. *Hum. Mol. Genet.* **15**, 2945–2954 (2006).

21. M. Fujioka, H. Mistry, P. Schedl, J. B. Jaynes, Determinants of chromosome architecture: Insulator pairing in cis and in trans. *PLOS Genet.* **12**, e1005889 (2016).

22. M. Fujioka, X. Wu, J. B. Jaynes, A chromatin insulator mediates transgene homing and very long-range enhancer-promoter communication. *Development* **136**, 3077–3087 (2009).

23. M. Galloni, H. Gyurkovics, P. Schedl, F. Karch, The bluetail transposon: Evidence for independent cis-regulatory domains and domain boundaries in the bithorax complex. *EMBO J.* **12**, 1087–1097 (1993).

24. A. T. Hark, C. J. Schoenherr, D. J. Katz, R. S. Ingram, J. M. Levorse, S. M. Tilghman, CTCF mediates methylation-sensitive enhancer-blocking activity at the H19/Igf2 locus. *Nature* **405**, 486–489 (2000).

25. F. Karch, M. Galloni, L. Sipos, J. Gausz, H. Gyurkovics, P. Sched, Mcp and Fab-7: Molecular analysis of putative boundaries of cis-regulatory domains in the bithorax complex of Drosophila melanogaster. *Nucleic Acids Res.* **22**, 3138–3146 (1994).

26. V. Narendra, M. Bulajic, J. Dekker, E. O. Mazzoni, D. Reinberg, CTCF-mediated topological boundaries during development foster appropriate gene regulation. *Genes Dev.* **30**, 2657–2662 (2016).

27. A. Sparago, F. Cerrato, M. Vernucci, G. B. Ferrero, M. C. Silengo, A. Riccio, Microdeletions in the human H19 DMR result in loss of IGF2 imprinting and Beckwith-Wiedemann syndrome. *Nat. Genet.* **36**, 958–960 (2004).

28. H. N. Cai, P. Shen, Effects of cis arrangement of chromatin insulators on enhancer-blocking activity. *Science* **291**, 493–495 (2001).

29. E. Muravyova, A. Golovnin, E. Gracheva, A. Parshikov, T. Belenkaya, V. Pirrotta, P. Georgiev, Loss of insulator activity by paired Su(Hw) chromatin insulators. *Science* **291**, 495–498 (2001).

30. Y. B. Schwartz, G. Cavalli, Three-dimensional genome organization and function in Drosophila. *Genetics* **205**, 5–24 (2017).

31. G. Fudenberg, M. Imakaev, C. Lu, A. Goloborodko, N. Abdennur, L.A. Mirny, Formation of chromosomal domains by loop extrusion. *Cell Rep.* **15**, 2038–2049 (2016).

32. A. L. Sanborn, S. S. Rao, S. C. Huang, N. C. Durand, M. H. Huntley, A. I. Jewett, I. D. Bochkov, D. Chinnappan, A. Cutkosky, J. Li, K. P. Geeting, A. Gnirke, A. Melnikov, D. McKenna, E. K. Stamenova, E. S. Lander, E. L. Aiden, Chromatin extrusion explains key features of loop and domain formation in wild-type and engineered genomes. *Proc. Natl. Acad. Sci. U.S.A.* **112**, E6456–6465 (2015).

33. P. J. Batut, X. Y. Bing, Z. Sisco, J. Raimundo, M. Levo, M. S. Levine, Genome organization controls transcriptional dynamics during development. *Science* **375**, 566–570 (2022).

34. K. P. Eagen, E. L. Aiden, R. D. Kornberg, Polycomb-mediated chromatin loops revealed by a subkilobase-resolution chromatin interaction map. *Proc. Natl. Acad. Sci. U.S.A.* **114**, 8764–8769 (2017).

35. Y. Ogiyama, B. Schuettengruber, G. L. Papadopoulos, J. M. Chang, G. Cavalli, Polycomb-dependent chromatin looping contributes to gene silencing during Drosophila development. *Mol. Cell* **71**, 73–88.e5 (2018).

36. M. J. Rowley, M. H. Nichols, X. Lyu, M. Ando-Kuri, I. S. M. Rivera, K. Hermetz, P. Wang, Y. Ruan, V. G. Corces, Evolutionarily conserved principles predict 3D chromatin organization. *Mol. Cell* **67**, 837–852.e7 (2017).

37. N. Nègre, C. D. Brown, P. K. Shah, P. Kheradpour, C. A. Morrison, J. G. Henikoff, X. Feng, K. Ahmad, S. Russell, R. A. H. White, L. Stein, S. Henikoff, M. Kellis, K. P. White, A comprehensive map of insulator elements for the *Drosophila* genome. *PLOS Genet.* **6**, e1000814 (2010).

38. Y. B. Schwartz, D. Linder-Basso, P. V. Kharchenko, M. Y. Tolstorukov, M. Kim, H.B. Li, A. A. Gorchakov, A. Minoda, G. Shanower, A. A. Alekseyenko, N. C. Riddle, Y. L. Jung, T. Gu, A. Plachetka, S. C.R. Elgin, M. I. Kuroda, P. J. Park, M. Savitsky, G. H. Karpen, V. Pirrotta, Nature and function of insulator protein binding sites in the Drosophila genome. *Genome Res.* **22**, 2188–2198 (2012).

39. M. Savitsky, M. Kim, O. Kravchuk, Y. B. Schwartz, Distinct roles of chromatin insulator proteins in control of the Drosophila bithorax complex. *Genetics* **202**, 601–617 (2016).

40. D. Chetverina, T. Aoki, M. Erokhin, P. Georgiev, P. Schedl, Making connections: Insulators organize eukaryotic chromosomes into independent cis-regulatory networks. *Bioessays* **36**, 163–172 (2014).

41. A. Simcox, S. Mitra, S. Truesdell, L. Paul, T. Chen, J. P. Butchar, S. Justiniano, Efficient genetic method for establishing Drosophila cell lines unlocks the potential to create lines of specific genotypes. *PLOS Genet.* **4**, e1000142 (2008).

42. T. I. Gerasimova, E. P. Lei, A. M. Bushey, V. G. Corces, Coordinated control of dCTCF and gypsy chromatin insulators in Drosophila. *Mol. Cell* **28**, 761–772 (2007).

43. D. Oliver, B. Sheehan, H. South, O. Akbari, C. Y. Pai, The chromosomal association/dissociation of the chromatin insulator protein Cp190 of Drosophila melanogaster is mediated by the BTB/POZ domain and two acidic regions. *BMC Cell Biol.* **11**, 101 (2010).

44. T. J. Parnell, E. J. Kuhn, B. L. Gilmore, C. Helou, M. S. Wold, P. K. Geyer, Identification of genomic sites that bind the Drosophila suppressor of Hairy-wing insulator protein. *Mol. Cell. Biol.* **26**, 5983–5993 (2006).

45. T. G. Kahn, E. Dorafshan, D. Schultheis, A. Zare, P. Stenberg, I. Reim, V. Pirrotta, Y.B. Schwartz, Interdependence of PRC1 and PRC2 for recruitment to polycomb response elements. *Nucleic Acids Res.* **44**, 10132–10149 (2016).

46. R. K. Maeda, F. Karch, The ABC of the BX-C: The bithorax complex explained. *Development* **133**, 1413–1422 (2006).

47. W. Bender, M. Akam, F. Karch, P. A. Beachy, M. Peifer, P. Spierer, E. B. Lewis, D. S. Hogness, Molecular genetics of the bithorax complex in Drosophila melanogaster. *Science* **221**, 23–29 (1983).

48. F. Karch, B. Weiffenbach, M. Peifer, W. Bender, I. Duncan, S. Celniker, M. Crosby, E. B. Lewis, The abdominal region of the bithorax complex. *Cell* **43**, 81–96 (1985).

49. E. B. Lewis, A gene complex controlling segmentation in Drosophila. *Nature* **276**, 565–570 (1978).

50. C. Iampietro, M. Gummalla, A. Mutero, F. Karch, R. K. Maeda, Initiator elements function to determine the activity state of BX-C enhancers. *PLOS Genet.* **6**, e1001260 (2010).

51. O. Kyrchanova, O. Maksimenko, A. Ibragimov, V. Sokolov, N. Postika, M. Lukyanova, P. Schedl, P. Georgiev, The insulator functions of the *Drosophila* polydactyl C2H2 zinc finger protein CTCF: Necessity versus sufficiency. *Sci. Adv.* **6**, eaaz3152 (2020).

52. P. V. Kharchenko, A. A. Alekseyenko, Y. B. Schwartz, A. Minoda, N. C. Riddle, J. Ernst, P. J. Sabo, E. Larschan, A. A. Gorchakov, T. Gu, D. Linder-Basso, A. Plachetka, G. Shanower, M. Y. Tolstorukov, L. J. Luquette, R. Xi, Y. L. Jung, R. W. Park, E. P. Bishop, T. K. Canfield, R. Sandstrom, R. E. Thurman, D. M. MacAlpine, J. A. Stamatoyannopoulos, M. Kellis, S. C. R. Elgin, M. I. Kuroda, V. Pirrotta, G. H. Karpen, P. J. Park, Comprehensive analysis of the chromatin landscape in Drosophila melanogaster. *Nature* **471**, 480–485 (2011).

53. Y. B. Schwartz, T. G. Kahn, P. Stenberg, K. Ohno, R. Bourgon, V. Pirrotta, Alternative epigenetic chromatin states of polycomb target genes. *PLOS Genet.* **6**, e1000805 (2010).

54. K. Van Bortle, E. Ramos, N. Takenaka, J. Yang, J. E. Wahi, V. G. Corces, Drosophila CTCF tandemly aligns with other insulator proteins at the borders of H3K27me3 domains. *Genome Res.* **22**, 2176–2187 (2012).

55. E. J. Kuhn-Parnell, C. Helou, D. J. Marion, B. L. Gilmore, T. J. Parnell, M. S. Wold, P. K. Geyer, Investigation of the properties of non-gypsy suppressor of hairy-wing-binding sites. *Genetics* **179**, 1263–1273 (2008).

56. Y. Zhang, T. Liu, C. A. Meyer, J. Eeckhoute, D. S. Johnson, B. E. Bernstein, C. Nusbaum, R. M. Myers, M. Brown, W. Li, X. S. Liu, Model-based analysis of ChIP-seq (MACS). *Genome Biol.* **9**, R137 (2008).

57. J. Dekker, K. Rippe, M. Dekker, N. Kleckner, Capturing chromosome conformation. *Science* **295**, 1306–1311 (2002).

58. E. Lieberman-Aiden, N. L. van Berkum, L. Williams, M. Imakaev, T. Ragoczy, A. Telling, I. Amit, B. R. Lajoie, P. J. Sabo, M. O. Dorschner, R. Sandstrom, B. Bernstein, M. A. Bender, M. Groudine, A. Gnirke, J. Stamatoyannopoulos, L. A. Mirny, E. S. Lander, J. Dekker, Comprehensive mapping of long-range interactions reveals folding principles of the human genome. *Science* **326**, 289–293 (2009).

59. V. E. Belozerov, P. Majumder, P. Shen, H. N. Cai, A novel boundary element may facilitate independent gene regulation in the Antennapedia complex of *Drosophila*. *EMBO J.* **22**, 3113–3121 (2003).

60. A. Golovnin, I. Biryukova, O. Romanova, M. Silicheva, A. Parshikov, E. Savitskaya, V. Pirrotta, P. Georgiev, An endogenous Su(Hw) insulator separates the *yellow* gene from the *Achaete-scute* gene complex in *Drosophila*. *Development* **130**, 3249–3258 (2003).

61. T. J. Parnell, M. M. Viering, A. Skjesol, C. Helou, E. J. Kuhn, P. K. Geyer, An endogenous suppressor of hairy-wing insulator separates regulatory domains in *Drosophila*. *Proc. Natl. Acad. Sci. U.S.A.* **100**, 13436–13441 (2003).

62. K. Zhao, C. M. Hart, U. K. Laemmli, Visualization of chromosomal domains with boundary element-associated factor BEAF-32. *Cell* **81**, 879–889 (1995).

63. Y. Dong, S. Avva, M. Maharjan, J. Jacobi, C. M. Hart, Promoter-proximal chromatin domain insulator protein BEAF mediates local and long-range communication with a transcription factor and directly activates a housekeeping promoter in *Drosophila*. *Genetics* **215**, 89–101 (2020).

64. C. Hou, L. Li, Z. S. Qin, V. G. Corces, Gene density, transcription, and insulators contribute to the partition of the Drosophila genome into physical domains. *Mol. Cell* **48**, 471–484 (2012).

65. M. I. Love, W. Huber, S. Anders, Moderated estimation of fold change and dispersion for RNA-seq data with DESeq2. *Genome Biol.* **15**, 550 (2014).

66. O. Kyrchanova, D. Chetverina, O. Maksimenko, A. Kullyev, P. Georgiev, Orientation-dependent interaction between *Drosophila* insulators is a property of this class of regulatory elements. *Nucleic Acids Res.* **36**, 7019–7028 (2008).

67. A. Kaushal, J. Dorier, B. Wang, G. Mohana, M. Taschner, P. Cousin, P. Waridel, C. Iseli, A. Semenova, S. Restrepo, N. Guex, E. L. Aiden, M. C. Gambetta, Essential role of Cp190 in physical and regulatory boundary formation. *Sci. Adv.* **8**, eabl8834 (2022).

68. S. V. Ulianov, E. E. Khrameeva, A. A. Gavrilov, I. M. Flyamer, P. Kos, E. A. Mikhaleva, A. A. Penin, M. D. Logacheva, M. V. Imakaev, A. Chertovich, M. S. Gelfand, Y. Y. Shevelyov, S. V. Razin, Active chromatin and transcription play a key role in chromosome partitioning into topologically associating domains. *Genome Res.* **26**, 70–84 (2016).

69. K. T. Chathoth, L. A. Mikheeva, G. Crevel, J. C. Wolfe, I. Hunter, S. Beckett-Doyle, S. Cotterill, H. Dai, A. Harrison, N. R. Zabet, The role of insulators and transcription in 3D chromatin organization of flies. *Genome Res.* **32**, 682–698 (2022).

70. N. E. Matthews, R. White, Chromatin architecture in the fly: Living without CTCF/cohesin loop extrusion?: Alternating chromatin states provide a basis for domain architecture in Drosophila. *Bioessays* **41**, e1900048 (2019).

71. C. Y. Pai, E. P. Lei, D. Ghosh, V. G. Corces, The centrosomal protein CP190 is a component of the gypsy chromatin insulator. *Mol. Cell* **16**, 737–748 (2004).

72. J. Vogelmann, A. le Gall, S. Dejardin, F. Allemand, A. Gamot, G. Labesse, O. Cuvier, N. Nègre, M. Cohen-Gonsaud, E. Margeat, M. Nöllmann, Chromatin insulator factors involved in long-range DNA interactions and their role in the folding of the Drosophila genome. *PLOS Genet.* **10**, e1004544 (2014).

73. A. Bonchuk, S. Denisov, P. Georgiev, O. Maksimenko, Drosophila BTB/POZ domains of "ttk group" can form multimers and selectively interact with each other. *J. Mol. Biol.* **412**, 423–436 (2011).

74. A. Bonchuk, O. Maksimenko, O. Kyrchanova, T. Ivlieva, V. Mogila, G. Deshpande, D. Wolle, P. Schedl, P. Georgiev, Functional role of dimerization and CP190 interacting domains of CTCF protein in *Drosophila melanogaster*. *BMC Biol.* **13**, 63 (2015).

75. S. Y. Kwon, V. Grisan, B. Jang, J. Herbert, P. Badenhorst, Genome-wide mapping targets of the metazoan chromatin remodeling factor NURF reveals nucleosome remodeling at enhancers, core promoters and gene insulators. *PLOS Genet.* **12**, e1005969 (2016).

76. E. Kravchenko, E. Savitskaya, O. Kravchuk, A. Parshikov, P. Georgiev, M. Savitsky, Pairing between *gypsy* insulators facilitates the enhancer action in *trans* throughout the *Drosophila* genome. *Mol. Cell. Biol.* **25**, 9283–9291 (2005).

77. H. B. Li, K. Ohno, H. Gui, V. Pirrotta, Insulators target active genes to transcription factories and polycomb-repressed genes to polycomb bodies. *PLOS Genet.* **9**, e1003436 (2013).

78. S. S. Rao, M. H. Huntley, N. C. Durnad, E. K. Stamenova, I. D. Bochkov, J. T. Robinson, A. L. Sanborn, I. Machol, A. D. Omer, E. S. Lander, E. L. Aiden, A 3D map of the human genome at kilobase resolution reveals principles of chromatin looping. *Cell* **159**, 1665–1680 (2014).

79. E. Yaffe, A. Tanay, Probabilistic modeling of Hi-C contact maps eliminates systematic biases to characterize global chromosomal architecture. *Nat. Genet.* **43**, 1059–1065 (2011).

80. R. Kumar, H. Sobhy, P. Stenberg, L. Lizana, Genome contact map explorer: A platform for the comparison, interactive visualization and analysis of genome contact maps. *Nucleic Acids Res.* **45**, e152 (2017).

81. A. R. Quinlan, I. M. Hall, BEDTools: A flexible suite of utilities for comparing genomic features. *Bioinformatics* **26**, 841–842 (2010).

82. J. C. Stansfield, K. G. Cresswell, M. G. Dozmorov, multiHiCcompare: Joint normalization and comparative analysis of complex Hi-C experiments. *Bioinformatics* **35**, 2916–2923 (2019).

83. J. M. Chang, Y. F. Weng, W. T. Chang, F. A. Lin, G. Cavalli, HiCmapTools: A tool to access HiC contact maps. *BMC Bioinformatics* **23**, 64 (2022).

84. J. Wolff, L. Rabbani, R. Gilsbach, G. Richard, T. Manke, R. Backofen, B. A. Grüning, Galaxy HiCExplorer 3: A web server for reproducible Hi-C, capture Hi-C and single-cell Hi-C data analysis, quality control and visualization. *Nucleic Acids Res.* **48**, W177–W184 (2020).

85. B. Langmead, S. L. Salzberg, Fast gapped-read alignment with Bowtie 2. *Nat. Methods* **9**, 357–359 (2012).

86. H. Li, B. Handsaker, A. Wysoker, T. Fennell, J. Ruan, N. Homer, G. Marth, G. Abecasis, R. Durbin, 1000 Genome Project Data Processing Subgroup, The sequence alignment/map format and SAMtools. *Bioinformatics* **25**, 2078–2079 (2009).

87. S. Althammer, J. Gonzalez-Vallinas, C. Ballare, M. Beato, E. Eyras, Pyicos: A versatile toolkit for the analysis of high-throughput sequencing data. *Bioinformatics* **27**, 3333–3340 (2011).

88. W. J. Kent, C. W. Sugnet, T. S. Furey, K. M. Roskin, T. H. Pringle, A. M. Zahler, D. Haussler, The human genome browser at UCSC. *Genome Res.* **12**, 996–1006 (2002).

89. A. Dobin, C. A. Davis, F. Schlesinger, J. Drenkow, C. Zaleski, S. Jha, P. Batut, M. Chaisson, T. R. Gingeras, STAR: Ultrafast universal RNA-seq aligner. *Bioinformatics* **29**, 15–21 (2013).

90. M. Imakaev, G. Fudenberg, R. P. McCord, N. Naumova, A. Goloborodko, B. R. Lajoie, J. Dekker, L. A. Mirny, Iterative correction of Hi-C data reveals hallmarks of chromosome organization. *Nat. Methods* **9**, 999–1003 (2012).

91. H. Moon, G. Filippova, D. Loukinov, E. Pugacheva, Q. Chen, S. T. Smith, A. Munhall, B. Grewe, M. Bartkuhn, R. Arnold, L. J. Burke, R. Renkawitz-Pohl, R. Ohlsson, J. Zhou, R. Renkawitz, V. Lobanenkov, CTCF is conserved from *Drosophila* to humans and confers enhancer blocking of the *Fab-8* insulator. *EMBO Rep.* **6**, 165–170 (2005).

92. T. I. Gerasimova, V. G. Corces, Polycomb and trithorax group proteins mediate the function of a chromatin insulator. *Cell* **92**, 511–521 (1998).

93. W. G. Whitfield, S. E. Millar, H. Saumweber, M. Frasch, D. M. Glover, Cloning of a gene encoding an antigen associated with the centrosome in Drosophila. *J. Cell Sci.* **89**, 467–480 (1988).

94. S. Cuartero, U. Fresan, O. Reina, E. Planet, M. L. Espinas, Ibf1 and Ibf2 are novel CP190-interacting proteins required for insulator function. *EMBO J.* **33**, 637–647 (2014).
